# Research on self-adaptive height adjustment control of shearer based on deep deterministic policy gradient

**DOI:** 10.1371/journal.pone.0329347

**Published:** 2026-01-22

**Authors:** Yadong Wang, Xuan Wang, Guocong Lin, Lijuan Zhao, Xunan Liu, Baoxuan Jia, Yuan Wang, Jingqiang He, Lianwei Ma

**Affiliations:** 1 School of Mechanical Engineering, Liaoning Technical University, Fuxin, China; 2 Liaoning Province Large Scale Industrial and Mining Equipment Key Laboratory, Fuxin, China; 3 Fuxin City Emergency Management Affairs Service Center, Fuxin, China; 4 Shanxi Tiandi Coal Mining Machinery Co., Ltd., Taiyuan, China; 5 National Engineering Laboratory for Coal Mining Machinery, Taiyuan, China; 6 China Coal Technology & Engineering Group Corporation, Taiyuan Research Institute Co., Ltd., Taiyuan, China; CINVESTAV IPN: Centro de Investigacion y de Estudios Avanzados del Instituto Politecnico Nacional, MEXICO

## Abstract

As a core component of the fully mechanized mining face, intelligent control of the shearer is fundamental to achieving unmanned mining and improving equipment reliability. To address the limitations of traditional optimization and deep reinforcement learning algorithms in achieving rapid and accurate self-adaptive control, this study proposes a novel shearer drum height control strategy based on the Deep Deterministic Policy Gradient (DDPG) algorithm. The 4602 workface at Yangcun Coal Mine and the MG2 × 55/250-BWD shearer model were used as engineering cases. A hybrid SVD-CWT and AlexNet transfer learning method was employed to identify coal and rock cutting states, achieving an accuracy of 95.06%. A DDPG-based self-adaptive hydraulic height adjustment model was then developed and validated through Matlab/Simulink and AMESim co-simulation, as well as a similarity-based physical test platform. Results show that the proposed method significantly outperforms conventional and fuzzy PID controls, reducing response time to 0.091 s and steady-state error to 0.00052 mm. Compared with TD3 and SAC algorithms, the system exhibited faster response, higher stability, and stronger anti-interference capability. The mean maximum error between simulation and experimental results was only 3.14%, confirming the feasibility and robustness of the proposed control strategy. This study provides a reliable approach for intelligent, adaptive height control of shearers under complex coal seam conditions.

## 1 Introduction

As a core piece of equipment in fully mechanized mining faces, intelligent control of the shearer lays the foundation for unmanned, intelligent mining operations. Due to geological activity, coal seam thickness often varies significantly across the mining face, requiring continuous adjustment of the shearer drum height along the coal-rock interface to maximize extraction rates and minimize rock cutting. Self-adaptive height adjustment of the shearer is therefore essential for effective intelligent control. This requires accurate real-time identification of coal and rock cutting states, using these states as a basis to adjust drum height dynamically in response to changes in coal seam thickness. This capability enables the front and rear drums to adaptively adjust height as the shearer moves forward, optimizing coal extraction while avoiding contact with the roof and floor. Consequently, achieving environment-self-adaptive drum height adjustment is a prominent focus in this research area, both domestically and internationally. Research in this field can be categorized into three main directions:① studying the height adjustment system’s performance through software simulation. Zhang et al. [1] conducted a co-simulation study on system dynamics using AMESim and ADAMS to analyze and improve the arm vibration phenomenon, implementing a PID-based electro-hydraulic proportional control system for precise drum height adjustment. Zhang et al. [2] used Automation Studio software to simulate the drum height adjustment process and examined the advantages and disadvantages of different neutral functions in the directional control valve, providing insights for the design of shearer hydraulic height adjustment systems.② Improving the height adjustment system’s performance through analysis of its mathematical model. Su et al. [3] analyzed the effects of cylinder stroke on the dynamic characteristics and stability of the hydraulic height adjustment system by developing a dynamic model for the shearer’s hydraulic height adjustment system. Ren et al. [4] proposed an optimized trajectory for memory cutting by developing a memory cutting model based on research into shearer posture and position tracking.③ Enhancing the height adjustment control system’s performance through improved control algorithms. References [5–9] have integrated optimization algorithms into traditional PID control strategies, forming modified PID control strategies. Comparative results with traditional control strategies indicate that this approach effectively addresses issues of slow response speed and low accuracy in shearer drum height adjustment control systems. Liu et al. [10] proposed a hybrid trajectory-tracking control strategy based on Linear Quadratic Regulator-Extended Boundary Conditions(LQR-EBC), which was validated experimentally for feasibility and performance advantages. In recent years, rapid advances in IOT, big data, AI, and 5G technology have provided advanced research tools for promoting intelligent, unmanned mining at fully mechanized faces. Liu et al [11] introduced an indirect self-adaptive prescribed performance control method using a novel neural network observer to achieve automatic height adjustment for the shearer. Additionally, Cui [12] developed an intelligent shearer height adjustment and speed control system based on 5G and cloud-edge collaborative technology, with industrial testing verifying its feasibility.

An analysis of the aforementioned literature reveals several limitations in previous approaches to studying the control performance of hydraulic height adjustment systems using classical control algorithms (such as conventional PID and fuzzy PID control). While enhancing the performance of hydraulic components can improve the overall performance of the hydraulic height adjustment system to a certain extent, it does not provide environmental adaptability and thus fails to meet the basic requirements of intelligent and unmanned mining. Traditional control algorithms also have drawbacks to varying degrees; for instance, conventional PID control can achieve good results when its parameters are properly tuned, but it struggles to maintain the original control effect and tracking accuracy under complex coal seam occurrence conditions, and parameter tuning is both difficult and low in precision [13]. Although PID controllers with parameters tuned via fuzzy controllers, neural networks, or optimization algorithms have improved adaptability to some extent, the dynamic adaptive adjustment of fuzzy rules and neural network parameters is difficult to achieve [14], resulting in a lack of self-learning and self-improvement capabilities, which limits their suitability for working conditions with complex occurrence conditions. Deep reinforcement learning algorithms, which continuously update their learning strategies based on rewards or penalties obtained from interactions with the environment, are more adaptable to changing environments and have been widely applied in fields such as UAV path planning, robot posture control, autonomous driving [15–23], achieving favorable control effects. Furthermore, In proton exchange membrane fuel cells, self-regulation methods have successfully addressed dynamic operating conditions [24]. Current typical deep reinforcement learning algorithms include DQN, DDPG, SAC, and TD3. However, the shearer’s hydraulic height adjustment system is a strongly nonlinear dynamic system with a continuous action space, requiring a balance between “control precision” and “real-time performance”. Therefore, determining an algorithm suitable for this system is particularly crucial. DQN struggles with large action spaces, especially continuous action scenarios, and has low sensitivity to environmental changes, making it unable to respond promptly to dynamic environmental changes [25]. The SAC algorithm introduces an entropy regularization mechanism to enhance exploration, encouraging the agent to try more actions by maximizing policy entropy, but this may also cause the policy to fall into a “suboptimal strategy” in complex coal seam conditions-over-exploring non-optimal actions and struggling to converge to a precise control strategy [26]. The TD3 algorithm addresses the “Q-value overestimation” issue in DDPG by introducing a dual Critic network and a delayed policy update mechanism. While this improves stability, it increases the number of network layers and parameter scale, leading to a significant increase in computational load and reduced computational efficiency, which severely affects the real-time performance of the adjustment process [27]. As one of the deep reinforcement learning algorithms, DDPG is a deep reinforcement learning algorithm that combines value iteration and policy iteration [28,29]. It can perform self-learning, self-tuning, and adaptive adjustment according to complex working conditions, while balancing the requirements of “control precision” and “real-time performance”.

To achieve self-adaptive height control for a shearer, it is essential to address the issue of identifying the coal-rock cutting state. Over the years, researchers domestically and internationally have proposed various coal-rock recognition methods, which can be categorized into the following types:① Radiographic detection, based on differences in radioactive element content between roof and floor rocks that result in distinct radiation energy and intensity levels, allowing for estimation of coal seam thickness [30]; ② Operational state detection, using cutting force signals, vibration signals, or cutting motor current to identify coal-rock interfaces [31–33]; ③ Acoustic emission and radar detection, which extract characteristic acoustic signals generated during coal-rock cutting for identification [34,35]; ④ Image recognition, including visible and infrared image processing [36–38]. However, these methods often struggle to achieve high recognition accuracy.

In response to this challenge, this study proposes a technique that denoises the time-domain vibration acceleration signals of the cutting drum using SVD-CWT, converts them to time-frequency spectrograms, and inputs them into an AlexNet transfer learning model to recognize the coal-rock cutting state. This recognition result serves as the basis for shearer height control, for which we propose a DDPG-based self-adaptive height control strategy. The feasibility and effectiveness of this control strategy are verified through system modeling, simulation analysis of control performance, co-simulation of control strategy, and physical testing, indicating that the strategy is highly suitable for self-adaptive height control systems in nonlinear, complex conditions with continuous action spaces. This approach is expected to advance intelligent shearer cutting control.

## 2 Theoretical background

### 2.1 The mathematical model of the height adjustment control system

**Fig 1:**
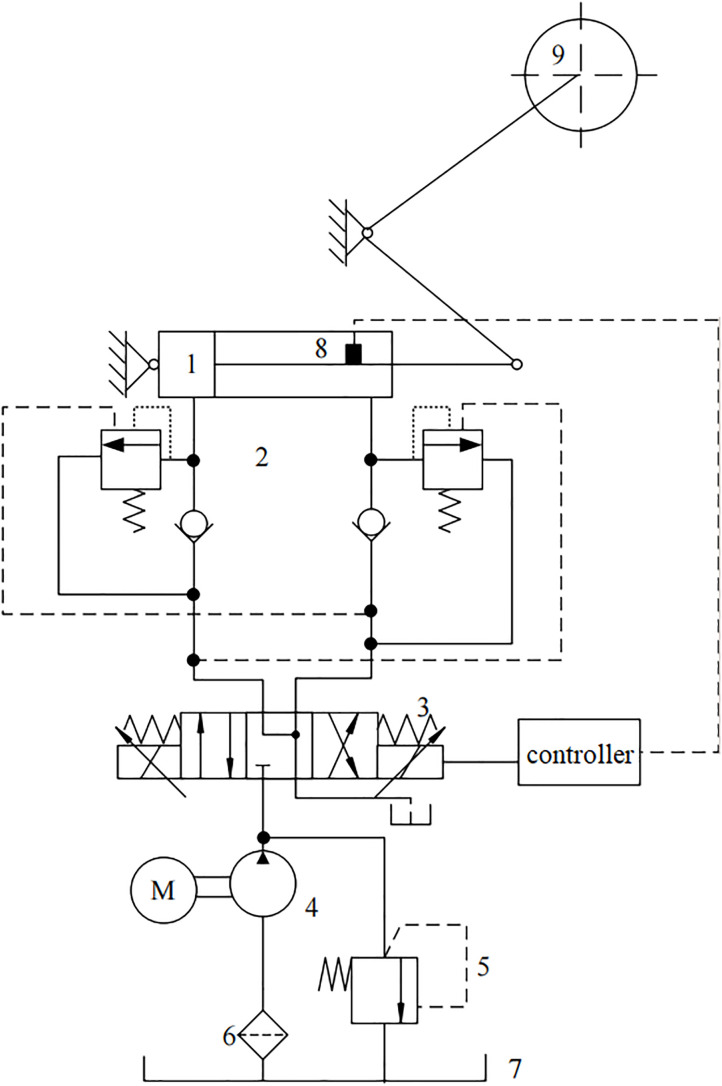
Structure diagram of the self-adaptive height adjustment hydraulic system of the shearer. 1-Height adjusting hydraulic cylinder; 2-balance valve; 3-electro-hydraulic proportional directional valve; 4-height adjusting oil pump; 5-overflow valve; 6-filter; 7-oil tank; 8-detecting device; 9-rocker arm.

The structure of the shearer’s self-adaptive height adjustment hydraulic system is shown in Fig 1.

**Fig 2:**
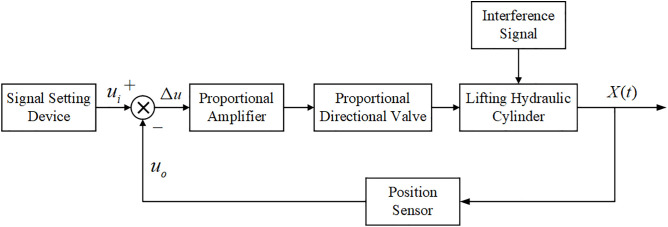
The block diagram of the height adjustment control system for the shearer.

The control system for increasing the electromechanical-hydraulic ratio in shearers typically consists of a proportional amplifier, electro-hydraulic directional valve, lifting hydraulic cylinder, interference signals, and signal detection and processing devices [8]. The system’s structure is illustrated in Fig 2.

The transfer functions of the components in the system shown in Fig 2 are determined based on the block diagram.

The proportional amplifier outputs a current that is proportional to the input voltage, and can be considered a proportional element:


$$G_{m}\left(S\right)=K_{m}$$ (1)

Where, $K_{m}$ is proportional amplification factor, A/V.

In engineering, the proportional direction valve is generally considered a second-order element, with the transfer function given by:


$$G_{v}\left(S\right)=\frac{K_{v}}{\frac{S^{2}}{{\omega_{v}}^{2}}+\frac{2\zeta_{v}}{\omega_{v}}s+1}$$ (2)

Where, $K_{v}$ is the flow gain of the proportional direction valve, $m^{\text{3}}\cdot s^{-1}\cdot A^{-1}$; $\omega_{v}$ is the natural frequency of the proportional direction valve, $rad\cdot s^{-1}$; $\zeta_{v}$ is the damping ratio of the proportional switching valve.

In engineering, when the elastic load is neglected, the actuator and controlled object are generally considered as a combination of an integrator and a second-order element, with the transfer function given by:


$$G_{h}\left(S\right)=\frac{K_{h}}{S\left(\frac{S^{2}}{{\omega_{h}}^{2}}+\frac{2\zeta_{h}}{\omega_{h}}s+1\right)}$$ (3)

Where, $K_{h}$ is the gain of the actuator hydraulic cylinder, Kh=1/1Ah\nulldelimiterspaceAh; $A_{h}$ is the effective working area of the hydraulic cylinder, m^2^, Depending on the direction of the piston rod movement, either the piston area $A_{1}$ or the annular area $A_{2}$ is considered.; $\zeta_{h}$ is the damping ratio of the hydraulic cylinder-load mass system; $\omega_{h}$ is the natural frequency of the hydraulic cylinder-load mass system, $rad\cdot s^{-1}$.

The input of the displacement sensor is the displacement signal of the hydraulic cylinder’s piston rod, and the output is a voltage signal fed back to the comparator. This can be simplified as a proportional element:


$$G_{f}\left(S\right)=K_{f}$$ (4)

Where, $K_{f}$ is the gain coefficient of the displacement sensor, V/m.

**Fig 3:**
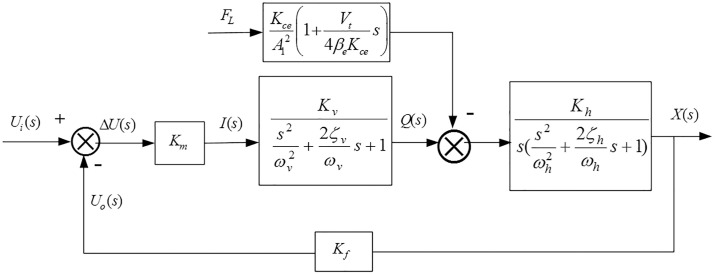
Transfer function block diagram of the self-adaptive height adjustment control system.

Based on the above analysis and in conjunction with reference [39], the transfer function block diagram of the system can be derived, as shown in Fig 3.

The rationality of the height adjustment system parameters and the validity of AMEsim-Simulink co-simulation support have been verified in the project team’s prior research [8], and thus are not elaborated herein. The simulation parameters for each component are selected as shown in Table 1, $F_{L}$ is the external disturbance force acting on the piston, kN; *V*_*t*_ is equivalent total volume, m^3^.

**Table 1:** Simulation parameters.

Parameter	Value
$A_{1}$	$2.0106\times 10^{-2}m^{2}$
$A_{2}$	$1.2252\times 10^{-2}m^{2}$
$\zeta_{h}$	0.7
$K_{m}$	2.25AV^-1^
$K_{f}$	6.56Vm^-1^
$K_{v}$	$4.8\times 10^{-4}m^{3}s^{-1}A^{-1}$
$\omega_{v}$	157rads^-1^
$\zeta_{v}$	0.2
$K_{ce}$	4.74×10−12m5/m5N·S\nulldelimiterspaceN·S
$\beta_{e}$	$0.9\times 10^{9}Pa^{2}$
$F_{L}$	5 kN
$\omega_{h}$	127.2rads^-1^
$\zeta_{h}$	0.1
$V_{t}$	$0.1803\times 10^{-2}m^{3}$

### 2.2 Geometric model of shearer height adjustment system

**Fig 4:**
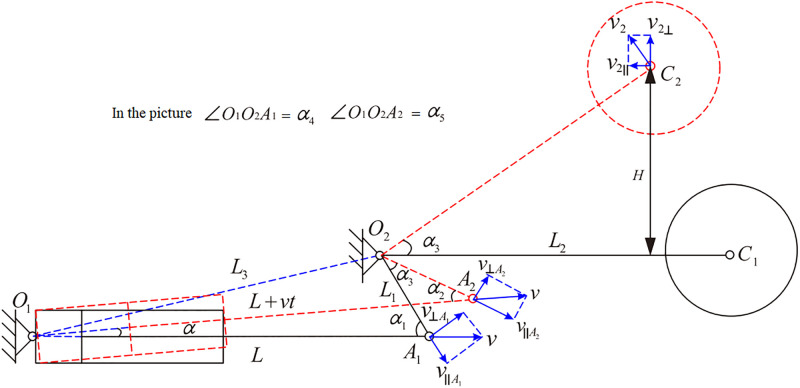
Simplified schematic diagram of shearer height adjustment hydraulic system.

A simplified diagram of the geometric structure of the shearer’s height adjustment system is shown in Fig 4. In this diagram, solid and dashed lines represent the drum’s two extreme positions: lowest and highest, respectively. Here, α is the adjustment cylinder angle at the two extreme positions, ($\backslash {(}^{\circ }$); *v* is the piston rod extension speed (mm/s); *L*_1_ is the length of the minor rocker arm (mm); *L*_2_ is the length of the major rocker arm (mm); *L*_3_ is the distance between the two hinge points (mm);the angles $\alpha_{1}$ and $\alpha_{2}$ are the angles between the piston and the minor rocker arm when the drum is at its two extreme positions ($\backslash {(}^{\circ }$); $\alpha_{3}$ is the rotation angle of the major and minor rocker arms, ($\backslash {(}^{\circ }$); $v_{⊥A_{1}}$, $v_{∥A_{1}}$, $v_{⊥A_{2}}$ and $v_{∥A_{2}}$ are the speed components of the piston rod extension *v* along and perpendicular to the minor rocker arm at the drum’s two extreme positions (mm/s); $v_{2}$, $v_{2∥}$ and $v_{2⊥}$ are the total movement speed of the drum, and its horizontal and vertical speed components (mm/s), respectively, when the drum is at its highest position; *H* is the maximum adjustable height of the drum in the vertical direction (mm).

Fig 4 shows that the vertical adjustment speed of the shearer drum height $v_{2⊥}$ is:


$$v_{2⊥}=v_{2}\cdot \cos\alpha_{3}$$ (5)

$$\frac{v_{2}}{L_{2}}=\frac{v_{⊥A_{2}}}{L_{1}}$$ (6)

From Equation (6), we obtain:


$$v_{2}=\frac{v_{⊥A_{2}}\cdot L_{2}}{L_{1}}$$ (7)

$v_{⊥A_{2}}$ is:


$$v_{⊥A_{2}}=v\cdot \sin\alpha_{2}$$ (8)

$$\sin\alpha_{2}=\sqrt{1-(\frac{{L_{1}}^{2}+{\left(L+vt\right)}^{2}-{L_{3}}^{2}}{2L_{1}\left(L+vt\right)})}$$ (9)

$$\alpha_{3}=\alpha_{5}-\alpha_{4}$$ (10)

$$\left\{ \begin{array}{c} \cos\alpha_{5}=\frac{{L_{1}}^{2}+{L_{3}}^{2}-{\left(L+vt\right)}^{2}}{2L_{1}L_{3}}\\ \cos\alpha_{4}=\frac{{L_{1}}^{2}+{L_{3}}^{2}-L^{2}}{2L_{1}L_{3}} \end{array}\right.$$ (11)

From equations (5)-(11), the relationship between the piston extension speed *v* and the vertical adjustment speed $v_{2⊥}$ of the shearer drum height can be derived as follows:


$$v_{2⊥}=\frac{L_{2}v}{L_{1}}\cdot \sqrt{1-\frac{{L_{1}}^{2}+{\left(L+vt\right)}^{2}-{L_{3}}^{2}}{2L_{1}\left(L+vt\right)}}\cdot \cos\left(\alpha_{5}-\alpha_{4}\right)$$ (12)

Where, $\alpha_{5}=\arccos\frac{{L_{1}}^{2}+{L_{3}}^{2}-{\left(L+vt\right)}^{2}}{2L_{1}L_{3}}$, $\alpha_{4}=\arccos\frac{{L_{1}}^{2}+{L_{3}}^{2}-L^{2}}{2L_{1}L_{3}}$.


$$H=\int \left(v_{2⊥}\right)=\frac{L_{2}\int v}{L_{1}}\cdot \sqrt{1-\frac{{L_{1}}^{2}+{\left(L+\int v\cdot t\right)}^{2}-{L_{3}}^{2}}{2L_{1}\left(L+\int v\cdot t\right)}}\cdot \cos\left(\alpha_{5}-\alpha_{4}\right)$$ (13)

Where, piston displacement: x=∫v_._

Using equations (12) and (13), the relationship between the extension and retraction displacement of the height adjusting hydraulic cylinder piston and the change in drum height can be determined.

### 2.3 Deep deterministic policy gradient algorithm

The core concept of Deep Reinforcement Learning (DRL) is to use deep neural networks to approximate the value and policy functions within reinforcement learning, with the objective of maximizing cumulative rewards to enable accurate environmental perception and precise control over the targeted system [40]. The Deep Deterministic Policy Gradient (DDPG) algorithm is a model-free DRL approach designed for continuous action spaces [41,42]. It achieves controller training and analysis solely based on the input-output data of the controlled system. The DDPG framework consists of an agent, rewards, an environment, an action space, and a state space. The agent comprises policy and learning algorithms, with its functions implemented through an Actor-Critic network structure. The Actor network models a function that maps control variables to actions, while the Critic network seeks an optimal policy by approximating the state-action value function $Q^{\pi }\left(s_{t},a_{t}\right)$. Using the Bellman equation, the Critic network calculates the cumulative reward for executing a specific action $a_{t}$ in a given state $s_{t}$.


$$Q^{\pi }\left(s_{t},a_{t}\right)=E_{r_{t},s_{t+1}\sim S}\left[r\left(s_{t},a_{t}\right)+\gamma E_{a_{t+1}\sim A}\left[Q^{\pi }\left(s_{t+1},a_{t+1}\right)\right]\right]$$ (14)

Where, *s*_*t*_ is the initial state; $s_{t+1}$ is the state at the next time interval; $a_{t}$ is a randomly chosen action taken in the initial state; $a_{t+1}$ is the optimal action taken in the subsequent state; $r\left(s_{t},a_{t}\right)$ is the immediate reward for an action $a_{t}$ taken in a given state $s_{t}$; γ is the discount factor; $Q^{\pi }\left(s_{t},a_{t}\right)$ is the state-action value function; E is the expected value.

Since the policy in DDPG is time-invariant, it can be expressed as: a=μ(s), Consequently, we have:


$$Q^{\mu }\left(s_{t},a_{t}\right)=E_{r_{t},s_{t+1}\sim S}\left[r\left(s_{t},a_{t}\right)+\gamma Q^{\mu }\left(s_{t+1},\mu \left(s_{t+1}\right)\right)\right]$$ (15)

The Critic network is parameterized using neural network weight parameters $\theta^{Q}$, and the loss function is defined based on mean squared error as follows:


$$L\left(\theta^{Q}\right)=E_{s_{t}\sim S}{,}_{a_{t}\sim A}{,}_{r_{t}\sim R_{t}}\left[{\left(y_{t}-Q\left(s_{t},a_{t}|\theta^{Q}\right)\right)}^{2}\right]$$ (16)

Here, the estimated value of the action-value function $y_{t}$ is:


$$y_{t}=r\left(s_{t},a_{t}\right)+\gamma Q^{\prime }\left(s_{t+1},\mu^{\prime }\left(s_{t+1}|\theta^{\mu^{\prime }}\right)|\theta^{Q^{\prime }}\right)$$ (17)

$\mu^{\prime }\left(s_{t+1}|\theta^{\mu^{\prime }}\right)$ 和$Q^{\prime }\left(s_{t+1},\mu^{\prime }\left(s_{t+1}|\theta^{\mu^{\prime }}\right)|\theta^{Q^{\prime }}\right)$ are the target networks of the Actor and Critic, respectively, using a soft target update strategy.


$$\theta^{Q^{\prime }}=\tau \theta^{Q}+\left(1-\tau \right)\theta^{Q^{\prime }}$$ (18)

$$\theta^{\mu^{\prime }}=\tau \theta^{\mu }+\left(1-\tau \right)\theta^{\mu^{\prime }}$$ (19)

Where, τ the momentum update rate.

Ultimately, the Critic network is updated by minimizing the loss function $L\left(\theta^{Q}\right)$, yielding an optimal policy evaluation value Q for assessing the action.

The Actor network is parameterized using neural network weight parameter $\theta^{\mu }$, and its iterative updates are performed using the sample gradient method as follows:


$$a=\mu \left(s|\theta^{\mu }\right)$$ (20)

$$\nabla_{\theta^{\mu }}\mu |s_{t}=E_{st\sim S}\left[{\nabla_{a}Q\left(s_{t},a|\theta^{Q}\right)|}_{a=\mu \left(s_{t}\right)}\nabla_{\theta_{\mu }}\mu \left(s_{t}|\theta^{\mu }\right)\right]$$ (21)

Where, $\nabla_{\theta^{\mu }}\mu |s_{t}$ is the policy gradient for updating the Actor network.

A policy noise mechanism is introduced to explore improved policies.


$$\mu^{\prime }\left(s_{t}\right)=\mu \left(s_{t}|\theta_{t}^{\mu }\right)+N_{t}$$ (22)

Where, $\mu^{\prime }\left(s_{t}\right)$ is the target policy obtained through exploration, $\mu \left(s_{t}|\theta_{t}^{\mu }\right)$ is the original Actor policy, $N_{t}$ is the policy noise.

During the agent’s search for the optimal policy, ‘State-Action’ pairs, represented as $\left(s_{t},a_{t},r_{t},s_{t+1}\right)$, are stored in the experience replay buffer. These pairs provide sample data for training and updating the Actor and Critic networks.

## 3 Proposed method

**Fig 5:**
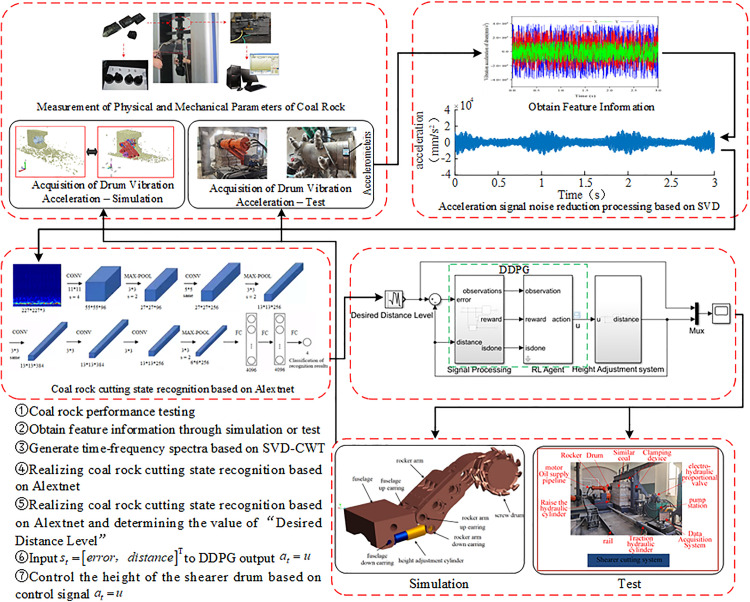
Technical route of the adaptive height adjustment control process for the shearer.

Taking the 4602 working face coal seam of the Yanzhou Coal Mining Group Yangcun Coal Mine and the MG2 × 55/250-BWD shearer spiral drum as the engineering objects, typical operating parameters were obtained. A method was proposed that utilizes Singular Value Decomposition (SVD) combined with Continuous Wavelet Transform (CWT) to denoise the vibration acceleration time-domain signals of the drum and convert them into time-frequency spectrograms, which are then input into the AlexNet transfer learning model for coal-rock cutting state recognition. Based on the recognition results, the current operating condition is determined, and the required displacement for adjusting the piston of the hydraulic cylinder is calculated using Equation (13). During this adjustment process, the Deep Deterministic Policy Gradient (DDPG) algorithm is employed as the controller for the self-adaptive height adjustment system of the shearer. A corresponding simulation environment, reward mechanism, and RL agent are designed. The DDPG controller continuously adjusts the system to minimize the error between the target and actual piston displacement, thereby achieving precise adjustment of the drum height. The technical route of the adaptive height adjustment control process for the shearer is shown in Fig 5.

### 3.1 Typical working condition technical parameters

**Fig 6:**
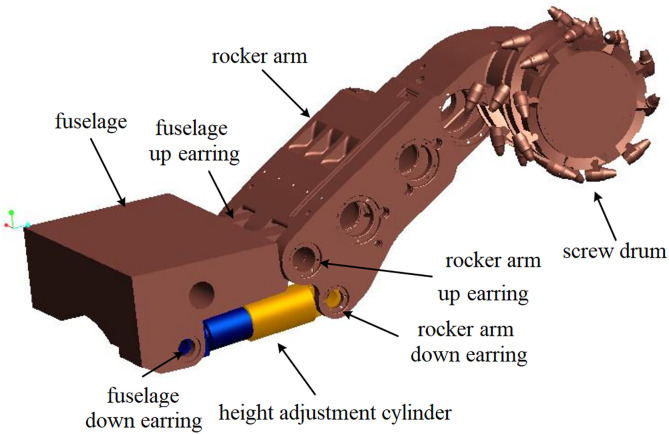
3D solid model of shearer height adjustment mechanism.

**Fig 7:**
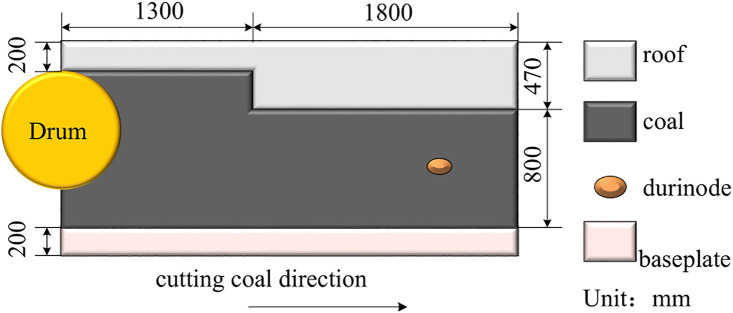
Coal wall model of working condition 4.

Self-adaptive height adjustment of the shearer is a critical measure to enhance production efficiency, prevent unnecessary energy consumption, and protect the equipment by adjusting the drum height based on coal-rock cutting state recognition. This study focuses on the MG2 × 55/250-BWD thin-seam shearer, whose model is shown in Fig 6, and main structural parameters are listed in Table 2. According to the shearer’s structure, the hydraulic cylinder piston has an extension range of 0–150 mm, enabling drum height adjustment within an 800 mm vertical range. The application scenario is based on the geological conditions of the 4602 working face at Yangcun Coal Mine operated by Yankuang Group, where the coal seam thickness ranges from 0.5 to 1.39 m. Durinode concretions are present in the seam, and, in some areas, coal seam sliding results in coal-rock faults, causing the drum to cut the roof layer, with random sliding magnitudes. Considering these conditions, this study analyzes and evaluates the performance of the self-adaptive height adjustment system for the shearer under four typical working conditions, as listed in Table 3. The relationship between the hydraulic cylinder piston retraction distance and the corresponding lowering height of the spiral drum can be calculated using the data in Table 2, in conjunction with Equations (12) and (13). Fig 7 illustrates the coal wall model under working condition 4.

**Table 2:** The main structural parameter values of the shearer and drum.

Drum Parameter	Value	Unit	Drum Parameter	Value	Unit
Drum Diameter	800	mm	Drum Hub Outer Diameter	465	mm
Spiral Blade Height	68	mm	Drum Hub Inner Diameter	425	mm
Spiral Blade Thickness	90	mm	Tooth Arrangement Type	Sequential	
Spiral Blade Pitch Angle	14	°	Drum Cutting Depth	630	mm
Number of Spiral Blades	2		Number of Teeth per Blade Line	2	
Length of Small Rock Arm				260	mm
Length of Drum Rock Arm				1400	mm
Distance from Lower Pivot of the Body to Upper Pivot of Rock Arm				712	mm
Distance from Drum’s Lowest Point to Hydraulic Cylinder Pivot				635	mm

**Table 3:** Typical working conditions.

Condition Number	Coal Wall Type	Hydraulic Cylinder Piston Retraction Distance (mm)	Spiral Drum Corresponding Downward Adjustment Height (mm)
1	Coal→Roof + Coal	20	100
2	Coal→Roof + Coal	40	214
3	Coal→Roof + Coal	60	325
4	Coal→Roof + Coal + Durinode	50	270

### 3.2 Identification of coal and rock cutting status

**Fig 8:**
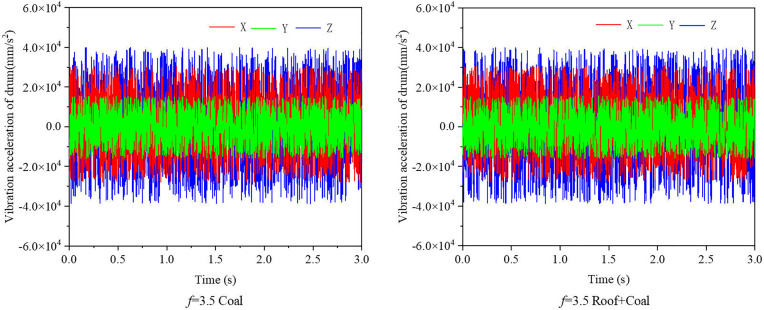
Vibration acceleration curves of the spiral drum under cutting conditions of *f* = 3.5 coal and *f* = 3.5 Roof +coal.

The vibration characteristics of the spiral drum vary with different operational conditions. This variation in vibration characteristics contains substantial information that can represent the properties of the cut coal-rock material. Based on the EDEM-RecurDyn coupled simulation technique, which was previously employed and validated by the project team to extract spiral drum vibration acceleration data [43], vibration acceleration signals from the spiral drum over a continuous 3 seconds period are used as coal-rock cutting state perception data. The sampling interval during the cutting process is 0.002 seconds. Under cutting conditions with a traction speed of 4.0 m/min and a drum speed of 90 r/min, the vibration acceleration curves in the directions of the drum’s centroid traction resistance (X), lateral force (Y), and cutting resistance (Z) are shown in Fig 8. The characteristic values of the vibration acceleration time-domain signals are listed in Table 4. From the values in Table 4, it is evident that the characteristic values of the time-domain vibration acceleration signals in all three directions are similar for both coal and coal-plus-roof cutting. The maximum, minimum, peak, and Root Mean Square (RMS) values have maximum variation rates of 3.19%, 2.62%, 4.94%, and 4.31%, respectively. This suggests that when the coal-rock material’s hardness coefficient is similar, relying solely on the time-domain response of vibration signals is insufficient for distinguishing between coal and coal-rock cutting states. Additionally, the time-domain signals are contaminated with redundant noise, which impacts the accuracy of coal-rock cutting state recognition. Therefore, a method based on Singular Value Decomposition (SVD) and Continuous Wavelet Transform (CWT) is proposed to denoise the vibration acceleration time-domain signals and convert them into time-frequency spectrograms. These spectrograms are then input into the AlexNet transfer learning model for coal-rock cutting state recognition [44].

**Table 4:** The signal characteristic value of vibration of drum X, Y and Z.

Coal-Rock Type	Direction	Vibration Acceleration Time-Domain Signal Features			
		Maximum Value (mm/s²)	Minimum Value (mm/s²)	Peak Value (mm/s²)	Root Mean Square (RMS) Value (mm/s²)
Coal	X	30833.40	−27985.46	6093.81	7839.54
	Y	15413.99	−16024.58	4095.37	1687.04
	Z	39874.25	−38761.63	9764.92	3782.99
Roof+Coal	X	31587.43	−28739.32	5883.71	7562.82
	Y	15921.43	−16239.43	3902.43	1617.32
	Z	40195.22	−39758.31	9597.41	3654.28

Singular Value Decomposition (SVD) is an effective method for feature extraction. The singular values obtained through decomposition can represent the intrinsic characteristics of data or signals, exhibiting strong stability and invariance. By truncating the singular value matrix, dimensionality reduction and compression can be achieved while retaining the primary information [45]. Based on SVD theory, the moving sliding-window method is applied to continuously segment the time-domain vibration acceleration signal of the helical drum, which is then transformed into a two-dimensional Hankel matrix ($A_{H}$). Its expression is given as:


$$A_{H}=\left[\begin{array}{c} \begin{array}{cccc} {}*20ca_{1} & a_{2} & \cdots & a_{n} \end{array}\\ \begin{array}{cccc} {}*20ca_{2} & a_{3} & \cdots & a_{n+1} \end{array}\\ \begin{array}{cccc} {}*20c\cdots & \cdots & \ddots & \cdots \end{array}\\ \begin{array}{cccc} {}*20ca_{m} & a_{m+1} & \cdots & a_{N} \end{array} \end{array}\right]$$ (23)

In this expression, *a*_*n*_ denotes the *n*-th collected vibration acceleration data point, measured in mm/s^2^. *N* represents the total number of collected vibration acceleration data points, where *N* = **n* + *m**-1. When *N* is even, n=N/2(m=N/2+1; when *N* is odd, n=(N+1)/2(m=(N+1)/2.

By performing a mathematical transformation on the matrix $A_{H}$, the following result is obtained:


$$A_{H}=USV^{T}$$ (24)

In this expression, $U\in R^{m\times m}$ and $V\in R^{n\times n}$ are the left and right singular orthogonal matrices, respectively, and S is a diagonal matrix, expressed as:


$$S=\left[\begin{array}{c} \begin{array}{cccc} {}*20c\lambda_{1} & & & \end{array}\\ \begin{array}{cccc} {}*20c & \lambda_{2} & & \end{array}\\ \begin{array}{cccc} {}*20c & & \ddots & \end{array}\\ \begin{array}{cccc} {}*20c & & & \lambda_{i} \end{array} \end{array}\right]$$ (25)

In this expression, $\lambda_{i}$ denotes the singular value, which satisfies $\lambda_{1}>\lambda_{2}>\cdots >\lambda_{i}$.

**Fig 9:**
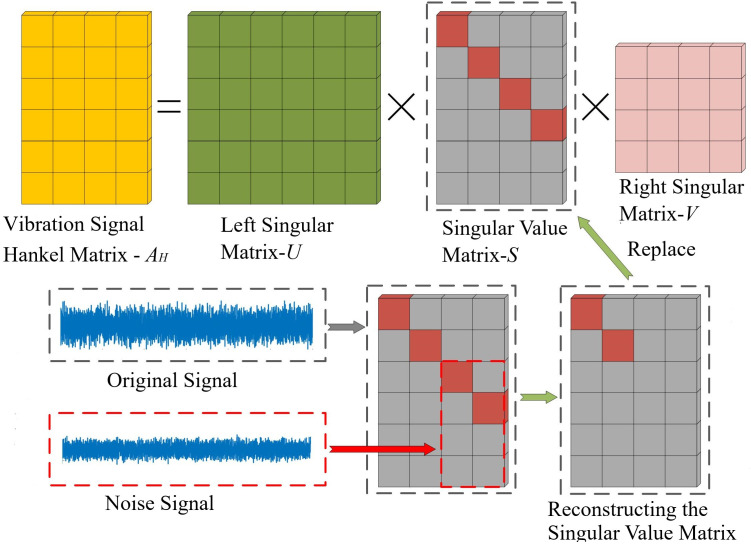
Basic principle of SVD denoising.

The magnitude of the singular values can indirectly reflect the degree of energy concentration. In general, significant singular values correspond to effective signals, whereas small singular values correspond to noise and interference. By selecting an appropriate number of singular values for data reconstruction, noise can be effectively eliminated. The principle is illustrated in Fig 9.

Determining the threshold that distinguishes noise from signal singular values is crucial for SVD-based denoising. Considering the characteristics of the helical drum vibration acceleration signals, the energy proportion method (EPM) is employed to determine the number of significant singular values. The core of this method is to calculate the proportion of each singular value’s squared magnitude relative to the total energy, with larger singular values corresponding to effective signals contributing more prominently to the total “energy.” The total energy of the vibration signal, the singular value energy proportion, and the cumulative energy proportion are determined based on the Frobenius norm, as shown in Equations (26)–(28). In this study, the energy threshold is set to 90%.

Total energy:


$${\left\|A_{H}\right\|}_{F}^{2}=\sum {\mathrm{\backslash nolimits}}_{i=1}^{k}\lambda_{i}^{2}$$ (26)

Energy proportion of singular values:


$$\eta_{j}=\frac{\lambda_{j}^{2}}{\sum {\mathrm{\backslash nolimits}}_{i=1}^{k}\lambda_{i}^{2}}$$ (27)

Cumulative energy proportion of the first *r* singular values:


$$\eta_{r}=\frac{\sum {\mathrm{\backslash nolimits}}_{j=1}^{r}\lambda_{j}^{2}}{\sum {\mathrm{\backslash nolimits}}_{i=1}^{k}\lambda_{i}^{2}}$$ (28)

**Fig 10:**
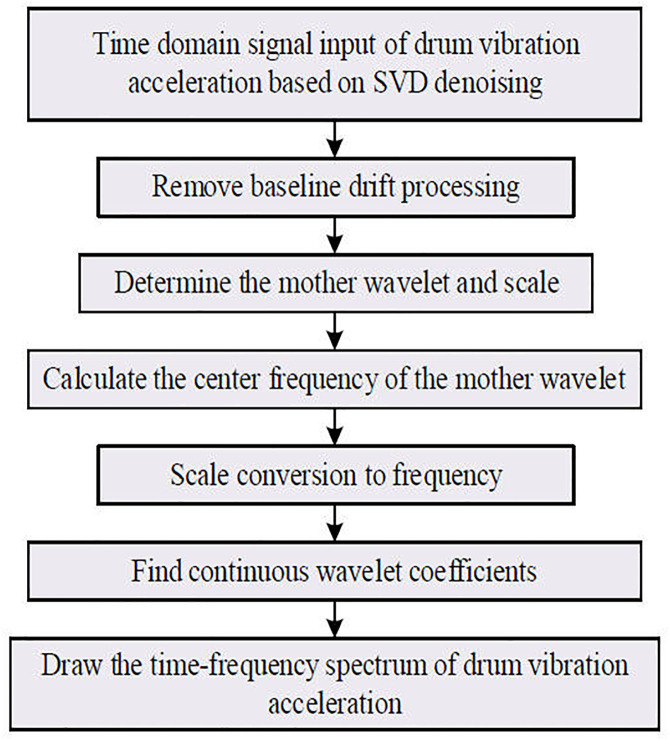
Generation of frequency spectrum of drum vibration acceleration.

**Fig 11:**
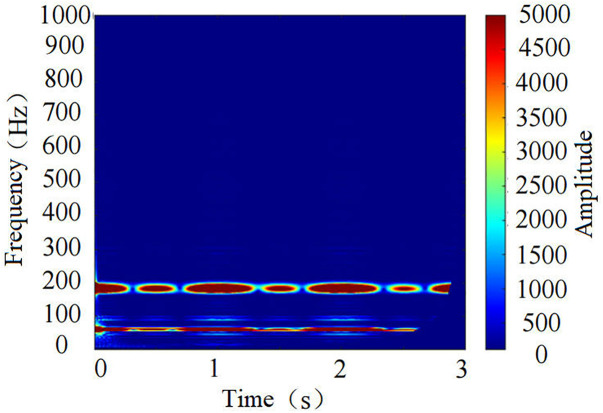
Time-frequency spectrogram under the *f* = 3.5 pure coal condition.

**Fig 12:**
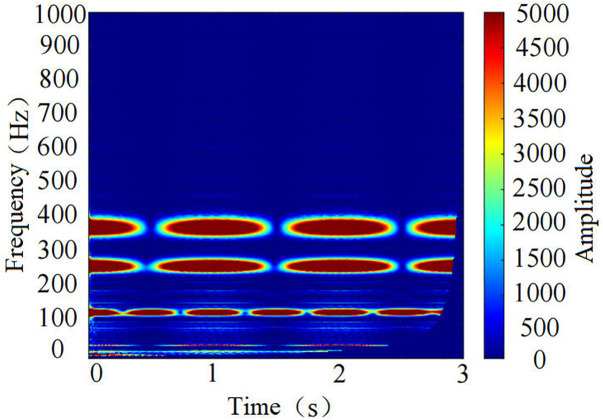
Time-frequency spectrogram under the *f* = 3.5 roof+coal condition.

The Continuous Wavelet Transform (CWT) is a time-frequency analysis method derived from the Fourier Transform (FT) and Short-Time Fourier Transform (STFT) theories. It uses a more suitable basis function for signal processing, making it particularly effective for non-stationary signals and self-adaptive to varying signal characteristics. The process of converting the denoised drum vibration acceleration time-domain signal into a time-frequency spectrogram using CWT is illustrated in Fig 10. The parameter settings of the CWT are shown in Table 5. The resulting time-frequency spectrograms based on CWT are shown in Figs 11 and 12.

**Table 5:** Parameter setting of CWT.

Parameter	Parameter selection
Wavelet Basis	cmor
Bandwidth	1
Center frequency	100
Scale	300
Pixel	227*227
Boundary treatment	symmetric extension

The time-frequency spectrograms reveal that under the *f* = 3.5 pure coal condition, the dominant frequencies are around 60 Hz and 180 Hz, with a narrow frequency band distribution. In contrast, under the **f* *= 3.5 roof+coal condition, the dominant frequencies are around 80 Hz, 170 Hz, and 240 Hz. Moreover, over time, the width and brightness of the frequency bands continuously change, indicating that the energy distribution and intensity of the two conditions differ. The time-frequency spectrograms contain rich varying features, allowing for a clear distinction between coal-rock cutting states.

**Fig 13:**
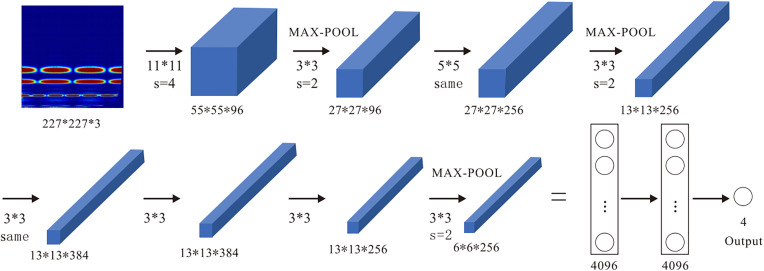
AlexNet transfer learning model.

As a typical convolutional neural network (CNN) model, AlexNet has been trained on 1.2 million images across 1000 categories, endowing it with robust image feature extraction capabilities. Reference [46] demonstrates that AlexNet achieved accurate recognition of characters on Qin bamboo slips, with a recognition accuracy as high as 99.89%. Reference [47] utilized AlexNet for real-time automatic recognition of plant leaves usable as livestock feed, yielding a recognition accuracy of 98.38%. Reference [48] conducted research on the recognition of crop disease images in complex backgrounds using AlexNet, and the results showed that the recognition accuracy was close to 90%. Additionally, Reference [49] pointed out that AlexNet is the most suitable deep neural network for coal-gangue separation. These studies provide theoretical guidance for the application of the AlexNet model in coal-rock cutting state recognition. However, considering the limited number of acquired time-frequency images, a network transfer learning technique is proposed to train model parameters with a small sample set. This technique has been proven feasible in References [50] and [51]. According to the classification of typical working conditions, the source domain and target domain are adapted by fine-tuning the network structure. The trained transfer network is employed as a feature extractor, and training is performed using time-frequency images obtained under different working conditions to adapt to the task of coal-rock cutting state recognition. The structure of the transfer learning model is illustrated in Fig 13, which mainly consists of an input layer, 5 convolutional layers, 3 pooling layers, 3 fully connected layers, and an output layer. In the transfer model, the convolution kernel sizes of CONV1 and CONV2 are 11 × 11 and 5 × 5, respectively, while those of CONV3, CONV4, and CONV5 are all 3 × 3. Shallow convolution utilizes large-dimensional kernels to extract shallow features of images, effectively reducing the image dimension and the number of parameters; deep convolution adopts small-dimensional kernels to extract more abstract deep features of images, with key parameter values listed in Table 6.

**Table 6:** Main parameter assignment.

Layer Structure	Input Channels	Output Channels	Input Feature Map Size	Output Feature Map Size	Kernel/Pool Size	Stride	Padding Value
Conv1	3	96	227 × 227	55 × 55	11 × 11	4	0
Max Pooling1	96	96	55 × 55	27 × 27	3 × 3	2	0
Conv2	96	256	27 × 27	27 × 27	5 × 5	1	2
Max Pooling2	256	256	27 × 27	13 × 13	3 × 3	2	0
Conv3	256	384	13 × 13	13 × 13	3 × 3	1	1
Conv4	384	384	13 × 13	13 × 13	3 × 3	1	1
Conv5	384	256	13 × 13	13 × 13	3 × 3	1	1
Max Pooling3	256	256	13 × 13	6 × 6	3 × 3	2	0

To quantitatively evaluate the recognition performance of the proposed AlexNet transfer learning model, the recognition accuracy (accuracy) is introduced as the performance metric. The specific calculation formula is given in Equation (29).


$$accuracy=\frac{TP+TN}{TP+TN+FP+FN}$$ (29)

In this equation, TP represents the number of true positives, FP represents false positives, FN represents false negatives, and TN represents true negatives.

To obtain hyperparameters that enable the AlexNet transfer learning network to achieve optimal recognition accuracy, and based on the selection of the training network solver as SGDM and Dropout set to 0.5 [52–53], the orthogonal experiment method is employed to analyze the effects of three factors—MaxEpochs, MiniBatchSize, and InitialLearnRate—on the model’s recognition accuracy. These three experimental factors are denoted as A, B, and C, respectively. According to the reasonable value ranges for each hyperparameter, a three-factor, four-level orthogonal experiment is conducted, and the factor-level table is presented in Table 7.

**Table 7:** Factor level table.

Level	A (MaxEpochs)	B (MiniBatchSize)	C (InitialLearnRate)
1	5	16	$1\times 10^{-2}$
2	10	32	$1\times 10^{-3}$
3	15	64	$1\times 10^{-4}$
4	20	128	$1\times 10^{-5}$

**Fig 14:**
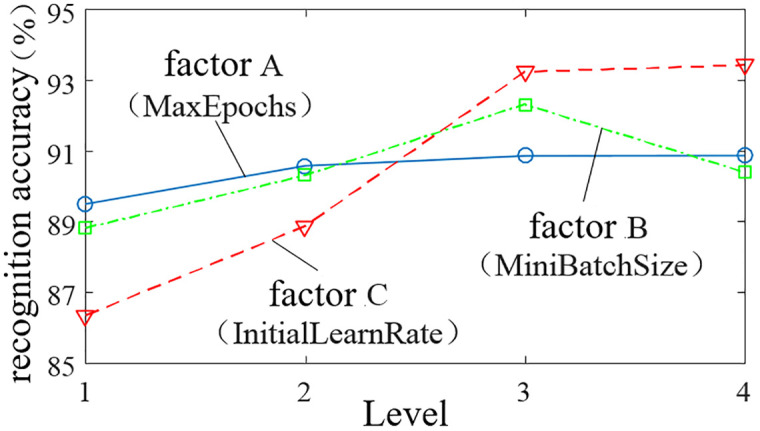
Factor trend analysis.

An $L_{16}\left(4^{3}\right)$ orthogonal table was selected to obtain 16 orthogonal experimental schemes, and the AlexNet transfer learning model was trained and tested using the training and testing datasets for each scheme. The characteristic values of the model’s recognition accuracy were calculated under each level of every factor. The experimental configuration schemes, orthogonal experiment results, and analysis of factor influence are presented in Tables 8 and 9. Using MaxEpochs, MiniBatchSize, and InitialLearnRate as the horizontal axes and the model recognition accuracy as the vertical axis, the influence trends of each factor on the model’s recognition accuracy were plotted, as shown in Fig 14.

**Table 8:** Test configuration scheme and orthogonal test results.

Test	Factor coding			Recognition accuracy (%)	Test	Factor coding			Recognition accuracy (%)
	A	B	C			A	B	C	
1	1	1	1	83.76	9	3	1	3	91.96
2	1	2	2	87.71	10	3	2	4	93.63
3	1	3	3	94.09	11	3	3	1	88.61
4	1	4	4	92.41	12	3	4	2	89.21
5	2	1	2	87.31	13	4	1	4	92.26
6	2	2	1	86.32	14	4	2	3	93.55
7	2	3	4	95.36	15	4	3	2	91.19
8	2	4	3	93.30	16	4	4	1	86.65

**Table 9:** Table of factor influence degree analysis.

	Recognition accuracy (%)		
	A	B	C
K1	357.97	355.29	345.34
K2	362.29	361.21	355.42
K3	363.41	369.25	372.90
K4	363.45	361.57	373.66
k1	89.49	88.81	86.34
k2	90.57	90.30	88.86
k3	90.85	92.31	93.23
k4	90.86	90.39	93.42
R	1.37	3.50	7.08
S	0.32	1.54	8.99

From the analysis of model recognition accuracy in Table 9, the results indicate R_C_ > R_B_ > R_A_, S_C_ > S_B_ > S_A_, showing that among the three factors—MaxEpochs, InitialLearnRate, and MiniBatchSize—InitialLearnRate has the most significant impact on model recognition accuracy, followed by MiniBatchSize, while MaxEpochs has the least influence. From the curve of factor A in Fig 14, the recognition accuracy initially increases and then stabilizes as MaxEpochs increases, eventually reaching 90.86%. The curve of factor B shows that recognition accuracy first increases and then decreases with increasing MiniBatchSize, achieving the maximum accuracy of 92.31% at level 3. The curve of factor C demonstrates that recognition accuracy continually improves with increasing InitialLearnRate, though the growth rate varies: when moving from level 1 to levels 2 and 3, accuracy rises significantly to 88.86% and 93.23%, respectively, while the increase from level 3 to level 4 is minimal, reaching 93.42%. Considering the above analysis and the need to reduce training time, the optimal hyperparameter combination is determined as MaxEpochs = 10, MiniBatchSize = 64, InitialLearnRate =$1\times 10^{-4}$, with sgdm as the solver and Dropout = 0.5.

Based on the constructed AlexNet network for transfer learning, the time-frequency spectrograms obtained under different operating conditions were divided into training and testing sets at a ratio of 4:1 for training and evaluation [54]. The recognition accuracy for the test samples was measured over five iterations, and the results are summarized in Table 10. As shown in Table 10, the average recognition accuracy reached 95.06%, which provides reliable data support for the precise control of the coal mining machine’s self-adaptive height adjustment.

**Table 10:** AlexNet network migration learning model recognition accuracy.

Experiment Number	Recognition Accuracy (%)	Average Recognition Accuracy (%)
1	95.33	95.06
2	94.97	
3	95.02	
4	95.51	
5	94.48	

To compare the performance superiority of the AlexNet transfer learning model, VGG-16, GoogLeNet, and AlexNet transfer learning models were selected for comparative experimental analysis. Based on the principle of controlled variables, the parameters of all network models were standardized. The recognition performance of each model was evaluated by the recognition accuracy on the training and testing datasets and the time consumed for recognition. The results are presented in Table 11.

**Table 11:** Recognition accuracy and recognition time under different models.

Model classification	Training set recognition accuracy (%)	Test set recognition accuracy (%)	The recognition time consumed (ms)
VGG-16	96.11	92.23	1884
GooleNet	96.34	91.04	1896
AlexNet	95.32	95.09	461

A comparison of the recognition accuracy data in Table 11 shows that the training set accuracy of the VGG-16 and GoogLeNet transfer learning models is slightly higher than that of the AlexNet transfer learning model, but the difference is minimal. However, the testing set recognition accuracies of VGG-16 and GoogLeNet are 2.86% and 4.05% lower than that of AlexNet, respectively. This outcome can be attributed to the deeper and wider network architectures of VGG-16 and GoogLeNet, which provide stronger feature extraction capabilities and superior performance on the training set. Nevertheless, the increased network depth significantly raises the number of parameters and complicates weight updates in shallow layers, resulting in decreased recognition accuracy. Additionally, deeper networks require larger datasets to avoid overfitting, and insufficient sample sizes can further degrade performance. Regarding recognition time, VGG-16 and GoogLeNet require substantially longer times than AlexNet due to the increased computational demands of their deeper structures. Overall, these analyses indicate that the AlexNet transfer learning model demonstrates stable recognition performance on both training and testing datasets while requiring less recognition time, thereby effectively enhancing the real-time capability of information transmission and processing.

### 3.3 Design of the self-adaptive height adjustment control for the shearer based on DDPG

**Fig 15:**
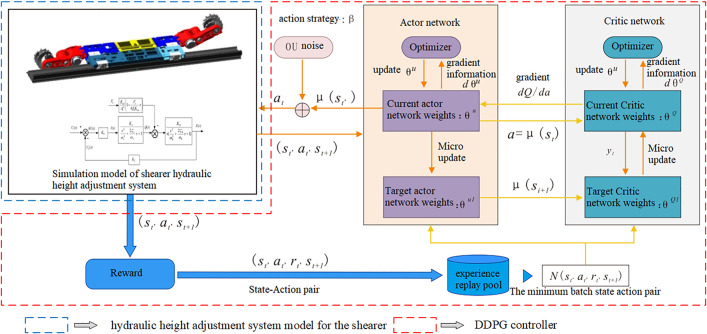
Algorithm architecture of the hydraulic height adjustment system for the shearer based on DDPG.

The algorithm architecture of the shearer hydraulic height adjustment system based on DDPG is shown in Fig 15. In order for the DDPG algorithm to learn the optimal control strategy that meets expectations, the design of the control process needs to be combined with the controlled object and control objectives. This includes the selection of action space and state space, the establishment of the height adjustment system Simulink model, the creation of the RL Agent model, and the design and selection of the reward function.

Guided by the algorithm architecture shown in Fig 15, a DDPG-based self-adaptive hydraulic height adjustment model for the shearer was built. This model continuously extracts the actual displacement of the hydraulic cylinder piston, compares it with the target displacement derived from the coal-rock cutting state recognition results, and calculates the error signal. Additionally, inspired by the concept of reducing steady-state error in a PID controller through historical tracking errors, the model uses the error (the difference between the actual and target displacement) at the current and previous time steps, along with the actual displacement value, as a 2D state space for the RL agent at each time step.


$$s_{t}={\left[error(distance\right]}^{T}$$ (30)

**Fig 16:**
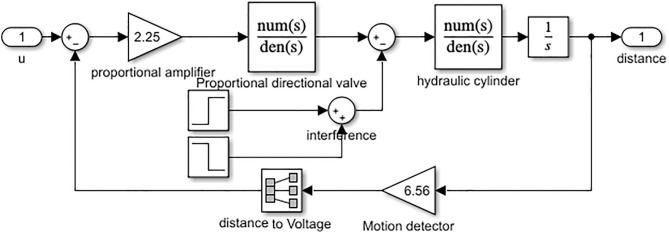
Simulink model of height adjustment system.

Based on the transfer function block diagram of the hydraulic height adjustment system, a Simulink model was constructed, as shown in Fig 16. The input is the voltage signal controlling the opening of the electro-hydraulic proportional valve, while the output is the displacement of the hydraulic cylinder piston.

For the hydraulic height adjustment system of the shearer, as shown in Fig 15, the control input is a voltage signal, Therefore, the action space for the RL agent is defined accordingly:


$$a_{t}=u$$ (31)

**Fig 17:**
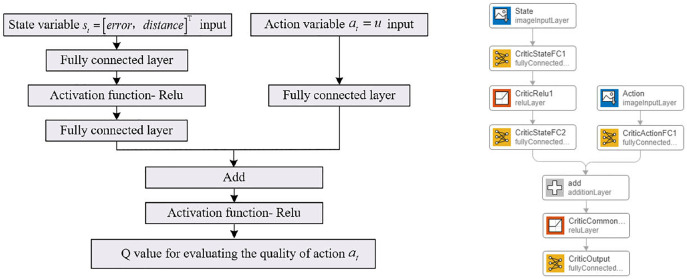
Deep neural network-Critic network.

**Fig 18:**
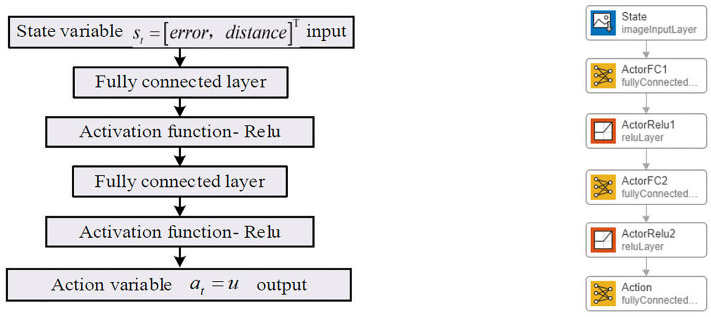
Deep neural network-Actor network.

The DDPG-based self-adaptive controller, implemented in the Simulink environment, requires control commands written in MATLAB files to interface with the neural network modules and build the RL agent. The RL agent should consist of a deep neural network with two inputs for simulating the Critic network, and a single-input, single-output deep neural network for simulating the Actor network, as shown in Figs 17 and 18.

The Critic network structure, as shown in Fig 17, includes two input layers: one for the state variable $a_{t}$ and the other for the action output variable *u*. The network consists of two hidden layers, with 100 and 50 neurons, respectively. The input layer for the action output variable is directly connected to the second hidden layer, and all hidden layers are fully connected. The output layer consists of a single neuron to evaluate the quality of actions, represented by the Q-value . All hidden layers use the Rectified Linear Unit (Relu) activation function.

The Actor network structure, as shown in Fig 18, includes an input layer for the state variable $a_{t}$ and two hidden layers. The number of neurons in the hidden layers matches that of the Critic network, and the hidden layers are fully connected. The output layer consists of a single neuron to represent the action output variable. Similar to the Critic network, all hidden layers use the Relu activation function. Details of the Critic/Actor networks and other parameters of the RL Agent are provided in Tables 12 and 13.

**Table 12:** Parameter setting of deep neural network.

	Learning Rate	Gradient Threshold Method	Gradient Threshold
Network Parameters Critic/Actor	$1\times 10^{-4}$	l2norm	1
	Optimizer	L2 Regularization Facto	Use Device
	Adam	$1\times 10^{-4}$	CPU

**Table 13:** Agent parameter settings.

Agent Parameters	Target Network Update Method	Target Network Update Delay Factor	Target Network Update Frequency	Noise Mechanism	
				Variance	Variance Decay Rate
Smoothing	$1\times 10^{-3}$	1	0.5	$1\times 10^{-5}$
Sampling Time	Reward Discount Factor	Batch Size	Experience Replay Pool Size	
0.05s	0.9	64	$1\times 10^{5}$	

The reward signal measures the agent’s contribution toward achieving the task goal. During training, the agent updates its policy based on the reward. By carefully designing the reward function, the controller’s performance can be enhanced, and the steady-state error in the adjustment process can be reduced. For the self-adaptive height adjustment control of the shearer, the reward function is defined and adjusted based on the error between the target and actual displacement of the hydraulic cylinder piston. Three types of reward functions-discrete, continuous, and hybrid-are designed, as shown in equations (32)-(33).

Discrete reward function $r_{1}$:


$$\begin{array}{c} r_{1}=0\left(\left|error\right|<0.1\right)-0.5\left(0.1\leq \left|error\right|<1\right)-1\left(1\leq \left|error\right|<5\right)-4\left(5\leq \left|error\right|<10\right)\\ \begin{array}{cc} {}*20c & \end{array}-7\left(10\leq \left|error\right|<17\right)-10\left(17\leq \left|error\right|<25\right)-15\left(\left|error\right|\geq 25\right) \end{array}$$ (32)

Where, *error* is between the actual and target displacement of the hydraulic cylinder piston, mm.

The discrete reward function divides the reward interval into seven sections: |error|<0.1, 0.1≤|error|<1, 1≤|error|<5, 5≤|error|<10, 10≤|error|<17, 17≤|error|<25, |error|≥25. As indicated in equation (30), when the error exceeds a specified range, the reward value becomes negative.

Continuous reward function $r_{2}$ is as follows:


$$r_{2}=-\left|error\right|$$ (33)

The continuous reward function is one that varies continuously with the hydraulic cylinder’s error value, where the reward decreases as the error increases.

Hybrid reward function $r_{3}$:


$$r_{3}=r_{1}+r_{2}$$ (34)

The hybrid reward function combines both discrete and continuous reward functions.

In the process of shearer, the core of the automatic adjustment of the drum height is the precise control of the hydraulic cylinder’s piston extension distance. By randomizing the reference value for the hydraulic cylinder’s piston extension distance using Equation (35), the agent is encouraged to periodically learn and continuously update its optimal control strategy to adapt to changing operational conditions.


distance=150*rand (35)

Where, rand is a random number between 0 and 1, 150 is the extension stroke of the hydraulic cylinder’s piston.

The DDPG-based self-adaptive lifting control model was trained using reward functions $r_{1}$, $r_{2}$ and $r_{3}$, to ensure that the training performance of the DDPG-based self-adaptive height adjustment control model meets the requirements of actual coal mining operations, the training termination condition was defined as follows: the steady-state error of the hydraulic cylinder piston displacement must remain below 0.5 mm for five consecutive training episodes, and the convergence behavior and speed were statistically analyzed, as shown in Table 14.

**Table 14:** Characteristic parameters of different reward functions.

Reward Function Type	Convergence	Training Episodes to Satisfy Termination Condition	Training Time(s)
Discrete Reward Function *r*_1_	Convergence	2460	5283.1
Continuous Reward Function *r*_2_	Convergence	1226	3057.1
Hybrid Reward Function *r*_3_	Convergence	542	1271.7

By comparing the characteristic parameters shown in Table 14, it is evident that the hybrid reward function exhibits a significantly faster convergence speed compared to the other two functions.

**Fig 19:**
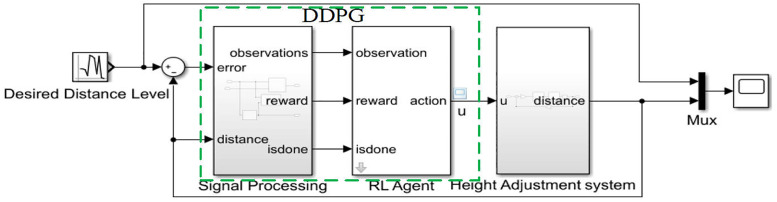
The DDPG-based self-adaptive hydraulic height adjustment system model for the shearer (Model I).

Based on the Simulink environment model of the height adjustment system and the DDPG controller model established in Fig 16, the final self-adaptive hydraulic height adjustment system model for the shearer based on DDPG (Model I) is obtained, as shown in Fig 19. In this model, the data for the Desired Distance Level comes from the target value of the height adjustment hydraulic cylinder piston displacement, which corresponds to the coal-rock cutting state recognition results.

**Fig 20:**
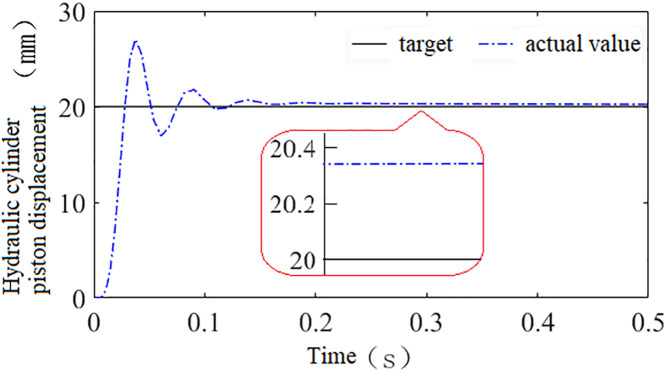
Shows the system’s control performance under reward function *r*_1_.

**Fig 21:**
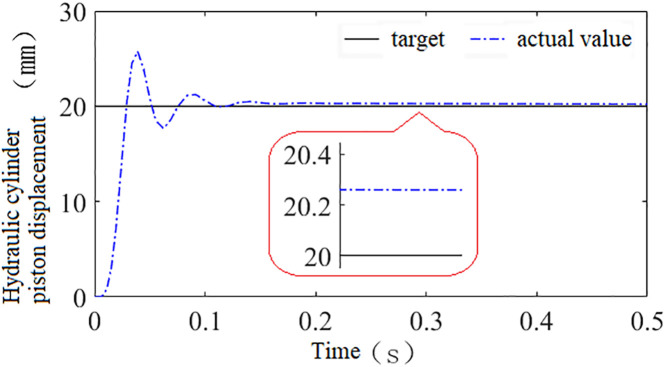
Shows the system’s control performance under reward function *r*_2_.

**Fig 22:**
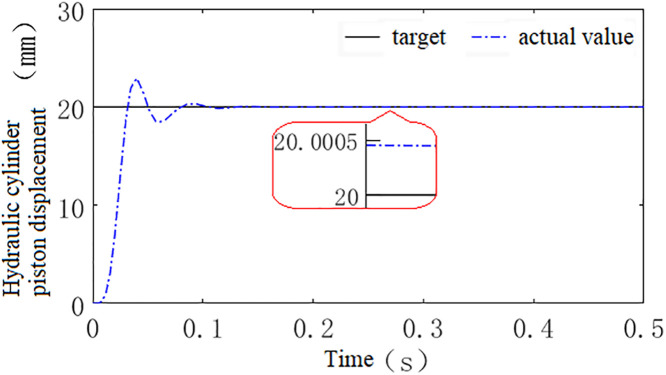
Shows the system’s control performance under reward function *r*_3_.

Using this model, a step signal with an amplitude of 20 is applied as the system input to compare the control performance of the hydraulic height adjustment system trained using the three types of reward functions, as shown in Figs 20–22.

Through comparative analysis, it is observed that the systems trained with discrete, continuous, and hybrid reward signals all exhibit fast response speeds. The time required to reach steady state is 0.16s, 0.12s, and 0.085s, respectively, while the steady-state errors are 0.33 mm, 0.26 mm, and 0.0005 mm. Compared to the systems trained with discrete and continuous reward signals, the system trained with the hybrid reward signal demonstrates superior performance in both speed and accuracy. Therefore, this paper selects the hybrid reward function as the training reward function for the agent.

## 4 System simulation and analysis

Using the established DDPG-based self-adaptive hydraulic height adjustment system model for the shearer (Model I), harmonic signals, square wave signals, and step signals with disturbances are employed to simulate the displacement variations of the hydraulic cylinder piston rod. The system’s tracking characteristics, anti-interference performance, environmental adaptability, and the control performance comparison of different algorithms are analyzed to evaluate the superiority of the DDPG control algorithm.

### 4.1 System tracking performance analysis

**Fig 23:**
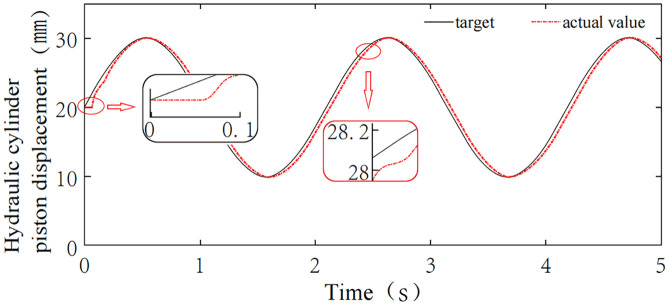
Tracking simulation of harmonic signal.

**Fig 24:**
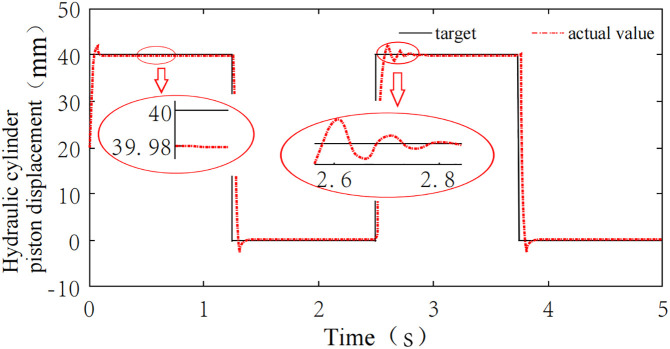
Tracking simulation of square wave signal.

The hydraulic cylinder piston displacement was simulated with harmonic signals of amplitude 10, offset 20, and angular velocity ω = 3 rad/s, and square wave signals with amplitude 40, period T = 2.5s, and duty cycle 50%. These signals were used to simulate continuous and abrupt changes in the piston displacement, in order to validate the tracking performance of the adaptive control system. A simulation time of 5 seconds was set. The simulation results are shown in Figs 23 and 24. From Fig 23, it can be seen that when the hydraulic cylinder piston displacement undergoes continuous changes simulated by a harmonic signal, the system tracking delay and steady-state error are 0.08s and 0.12 mm, respectively. Fig 24 shows that when the hydraulic cylinder piston displacement experiences abrupt changes simulated by a square wave signal, the system steady-state error is only 0.02 mm when the displacement remains constant. When the displacement undergoes a sudden change at 1.25s, the system responds rapidly and re-enters steady state after approximately 0.158s. Overall, the system can effectively track different input signals with a quick response and small steady-state error, demonstrating good tracking performance.

### 4.2 System anti-interference analysis

**Fig 25:**
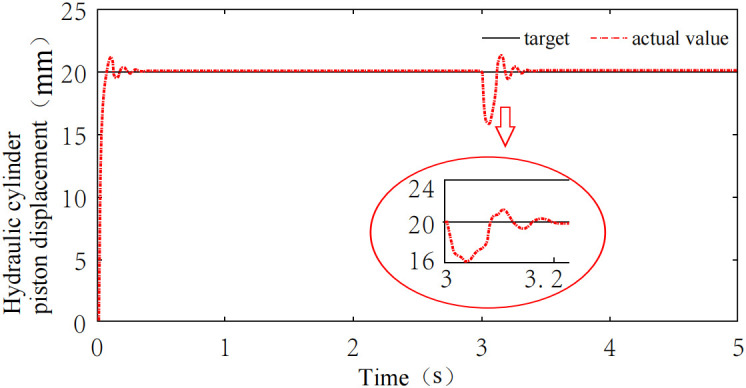
Simulation analysis under disturbance condition.

The height adjustment system must maintain strong anti-interference capability in the event of sudden external disturbances. To validate the system’s anti-interference performance, a disturbance is simulated to reflect the sudden loading change encountered when the coal cutter unexpectedly encounters pyrite nodules during the coal-rock cutting process. A step signal with an amplitude of 5 kN is added at the 3s mark as a disturbance signal, with the system response shown in Fig 25. Following the disturbance, the system reacts immediately and returns to a steady state within 0.13s, indicating a high level of anti-interference capability. Additionally, minimal oscillation is observed, demonstrating the system’s stable performance under such conditions.

### 4.3 System environmental self-adaptability analysis

**Fig 26:**
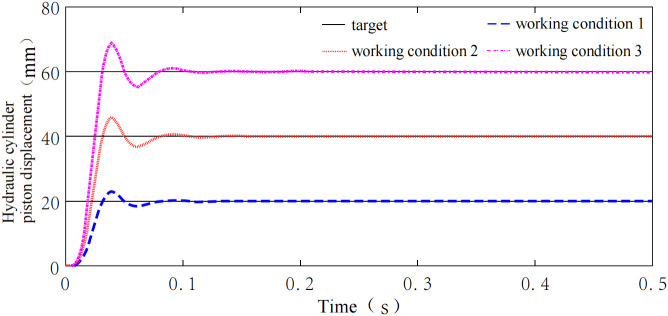
Environmental self-adaptability simulation analysis.

To verify the environmental self-adaptability of the height adjustment system, as well as its self-learning and self-improvement capabilities during training, simulations were conducted under three typical conditions presented in Table 3. Step signals with amplitudes of 20, 40, and 60 were applied to simulate the variation in hydraulic cylinder piston rod displacement required for each condition. The system response curves are shown in Fig 26. As illustrated, the times needed to reach steady state for step responses with amplitudes of 20, 40, and 60 were 0.085s, 0.08s, and 0.092s, respectively, with steady-state errors of 0.005 mm, 0.0046 mm, and 0.0055 mm. The time required to reach steady state and the steady-state errors are nearly identical across different conditions, with relative steady-state errors of 0.025%, 0.012%, and 0.009%. These results indicate that the system exhibits strong environmental self-adaptability and effective self-learning and self-improvement capabilities under varying conditions.

### 4.4 Comparision and validation analysis of algorithms

To evaluate the effectiveness of the DDPG-based self-adaptive height control for the shearer proposed in this study, a comparative analysis was conducted against conventional control algorithms and typical deep reinforcement learning algorithms.

**Fig 27:**
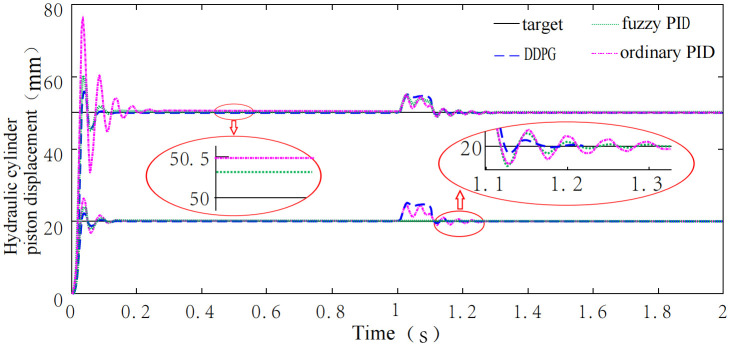
Comparison of control effects between DDPG and classical controllers.

Using the shearer self-adaptive height control system with conventional PID control, fuzzy PID control, and DDPG control, simulations were conducted to analyze the control performance of each method. Step signals with amplitudes of 20 and 50 were applied as system inputs. To simulate cutting through a hard durinode, a disturbance signal with an amplitude of 3 and a duration of 0.1s was introduced at 1s into the simulation. The results comparing the control performance of these three methods are presented in Fig 27.

Fig 27 shows that, when a step signal with an amplitude of 20 is applied, the systems controlled by conventional PID, fuzzy PID, and DDPG controllers reach steady-state in 0.135s, 0.11s, and 0.085s, respectively, all demonstrating good response speeds. The steady-state errors for each controller are 0.11 mm, 0.07 mm, and 0.0005 mm, respectively, with the DDPG controller achieving the highest control accuracy. When the step signal amplitude is increased to 50, without manual adjustment to controller parameters, the systems reach steady-state in 0.254s, 0.12s, and 0.091s, respectively, with steady-state errors of 0.48 mm, 0.27 mm, and 0.00052 mm. The DDPG controller again provides the best control performance, largely due to its adaptive capability, which is absent in the fixed-parameter design of the conventional PID controller.

Under a 0.1s disturbance signal applied at the 1s mark, the system controlled by the conventional PID and fuzzy PID controllers returned to steady-state in 1.27s and 1.22s, respectively. In contrast, the DDPG-controlled system required only 0.52s to stabilize, significantly reducing the adjustment time due to the limitations of conventional PID and fuzzy PID controllers in adapting to sudden disturbances.

In addition, the DQN, SAC, and TD3 controllers were trained using the same parameters as the DDPG controller. The shearer adaptive height adjustment system controlled by these four controllers was then simulated, with a step signal of amplitude 20 applied as the system input, and a disturbance signal of amplitude 3 and duration 0.1 s introduced at 1 s of simulation time. Performance metrics such as rise time, steady-state adjustment time, steady-state error, and time to recover from disturbance were analyzed, Based on the above analysis, the control performance of the six methods is evaluated, The results are shown in Table 15.

**Table 15:** Comparison of control performance of DDPG and other algorithms.

Algorithm	Performance Metric			
	Rise Time (s)	Adjustment Time (s)	Steady-State Error (mm)	Recovery Time after Disturbance (s)
Conventional PID	0.037	0.135	0.11	1.27
Fuzzy PID	0.039	0.11	0.07	1.22
DQN	0.038	0.093	0.0014	0.92
SAC	0.043	0.079	0.0824	0.55
TD3	0.091	0.138	0.0004	1.13
DDPG	0.041	0.085	0.0005	0.52

As shown in Table 15, when a step input signal with an amplitude of 20 is applied, the rise times of the system under conventional PID, fuzzy PID, DQN, SAC, TD3, and DDPG controllers are 0.037 s, 0.039 s, 0.038 s, 0.043 s, 0.091 s, and 0.041 s, respectively. Only the TD3-controlled system exhibits a significantly longer rise time, which can be attributed to the greater number of layers and hyperparameters in the TD3 deep neural network, resulting in higher computational demand. The rise times for conventional PID, fuzzy PID, DQN, SAC, and DDPG controllers are comparable. The settling times for these controllers are 0.135 s, 0.11 s, 0.093 s, 0.079 s, 0.138 s, and 0.085 s, respectively. Notably, DDPG reduces the adjustment time by 66.5% compared with conventional PID and by 38.4% compared with TD3, slightly exceeding SAC (0.079 s) in speed, while achieving a steady-state error of 0.0005 mm, significantly better than SAC (0.0824 mm). The steady-state errors for the six controllers are 0.11 mm, 0.07 mm, 0.0014 mm, 0.0824 mm, 0.004 mm, and 0.0005 mm, respectively. Among the deep reinforcement learning algorithms, SAC exhibits a larger steady-state error due to its tendency to converge to suboptimal policies when searching for the optimal control strategy [26]. The steady-state error of DDPG is only 0.1% of that of conventional PID and 0.18% of fuzzy PID, comparable to TD3 (0.0004 mm), while its disturbance recovery time (0.52 s) is only 46% of that of TD3. After disturbance, the adjustment times for the six controllers are 1.27 s, 1.22 s, 0.92 s, 0.55 s, 1.13 s, and 0.52 s, respectively. Among the deep reinforcement learning algorithms, DQN and TD3 exhibit longer adjustment times under disturbance, because DQN is suitable for discrete systems and TD3 involves substantial computation. In contrast, DDPG’s adjustment time under disturbance is only 40.9% of conventional PID and 42.6% of fuzzy PID, comparable to SAC (0.55 s), while achieving a steady-state error of 0.0005 mm, only 0.6% of SAC’s error (0.0824 mm). Overall, the system controlled by the DDPG controller demonstrates superior comprehensive performance compared with systems controlled by conventional PID, fuzzy PID, DQN, SAC, and TD3 controllers.

## 5 Feasibility verification of height control strategy based on AMESim-simulink co-simulation

**Fig 28:**
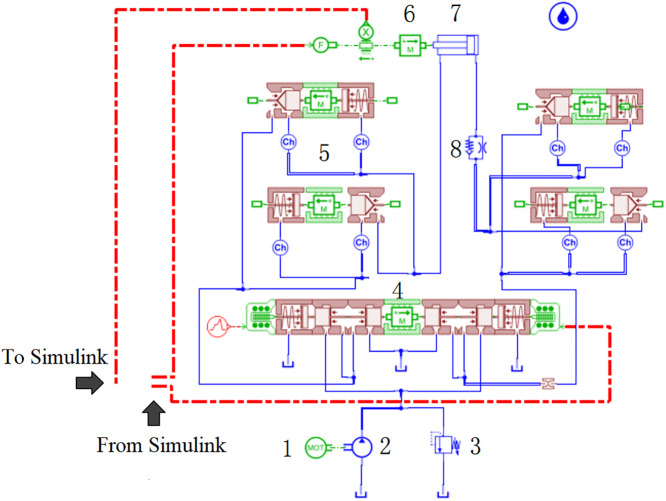
The AMEsim model of the hydraulic system of the shearer electro-hydraulic proportional height adjustment.

**Fig 29:**
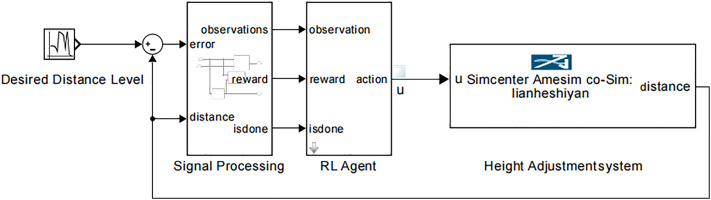
Self-adaptive hydraulic height adjustment model of shearer based on DDPG (Model II).

The electro-hydraulic proportional height control system models established using classical control theory primarily simulate linear time-invariant systems. However, the self-adaptive height adjustment process in shearers is subject to external influences such as geological conditions, which results in a nonlinear and time-varying behavior. To address the limitations of classical control theory in accurately modeling the dynamic performance of hydraulic systems and to make the research more reflective of engineering realities, a hydraulic height control system model was developed in the AMESim environment, as shown in Fig 1. By integrating AMESim and Simulink through an interface, the transfer-function-based model from the height control model in Fig 28 was replaced by the AMESim model shown in Fig 28, yielding a nonlinear, time-varying DDPG-based self-adaptive hydraulic height control system model (Model II) for shearers, as illustrated in Fig 29.

**Fig 30:**
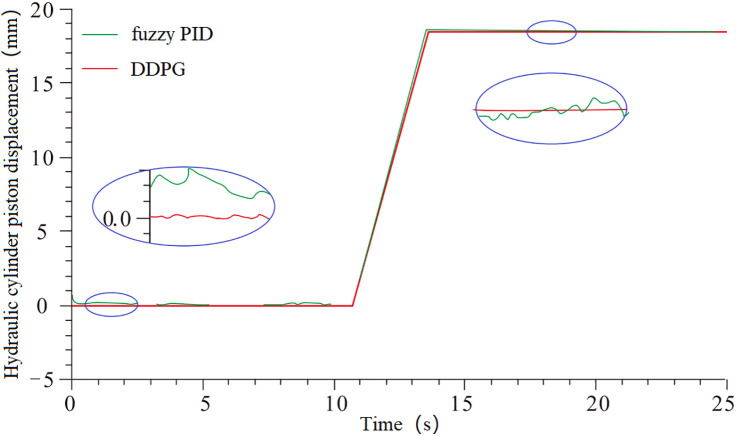
Piston displacement tracking and error.

**Fig 31:**
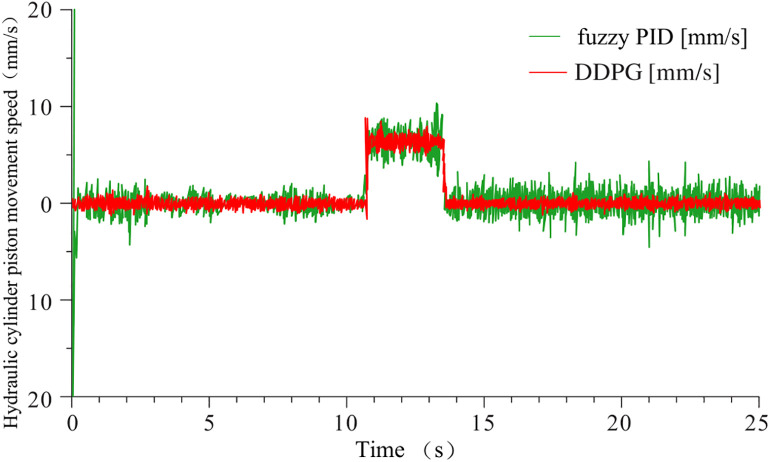
Piston motion speed.

To verify the feasibility of controlling the nonlinear and time-varying system, simulations were conducted using both the fuzzy PID controller and the DDPG controller on the model shown in Fig 29. The piston displacement tracking performance, steady-state error, and piston velocity were extracted to analyze the control effects of both methods. The results are shown in Figs 30 and 31.

From the data presented in Figs 30 and 31, it is evident that the systems controlled by fuzzy PID and DDPG exhibit different performance characteristics. In the pre-lift steady state, the displacement steady-state errors for the fuzzy PID and DDPG controllers are 0.2 mm and 0.0018 mm, respectively, with the latter showing more stable piston motion speed. During the lifting phase, the steady-state errors for the piston displacement are 0.32 mm for fuzzy PID and 0.002 mm for DDPG. Compared to the pre-lift steady phase, the fuzzy PID-controlled system exhibits a significant increase in piston motion speed fluctuations, whereas the DDPG-controlled system shows minimal variation, indicating superior self-adaptability of the latter.

Based on this analysis, simulations and control experiments were conducted using the DQN, SAC, TD3, and DDPG controllers on the model shown in Fig 29. The steady-state error and adjustment time for the hydraulic cylinder piston displacement were extracted, and the results are presented in Table 16.

**Table 16:** Comparison of control performance between DDPG and typical deep reinforcement learning algorithms in the joint simulation environment.

Algorithm	Performance Indicators	
	Steady-State Error (mm)	Adjustment Time (s)
DQN	0.0093	0.104
SAC	0.1027	0.087
TD3	0.0018	0.172
DDPG	0.0021	0.093

As shown in Table 16, the steady-state errors in the piston displacement of the hydraulic cylinder for the DQN, SAC, TD3, and DDPG controllers are 0.0093 mm, 0.1027 mm, 0.0018 mm, and 0.0021 mm, respectively. The adjustment times for each controller are 0.104s, 0.087s, 0.172s, and 0.093s, respectively. The performance trends of the four controllers in the adjustment process are consistent with the analysis in Table 15, with slight increases in the values due to the additional data transmission and computation time involved in the co-simulation process.

Based on the analysis above, compared to classical control algorithms (fuzzy PID) and typical deep reinforcement learning algorithms (DQN, SAC, TD3), the DDPG control strategy clearly demonstrates superior performance. It possesses the capabilities of self-learning, self-tuning, and self-adaptive. Furthermore, the DDPG-based control strategy exhibits rapid response and small steady-state errors, making it suitable for the adaptive height self-adjustment of shearers under complex operating conditions, contributing to intelligent and efficient coal mining.

## 6 Physical experiment verification

### 6.1 Experimental validation

**Fig 32:**
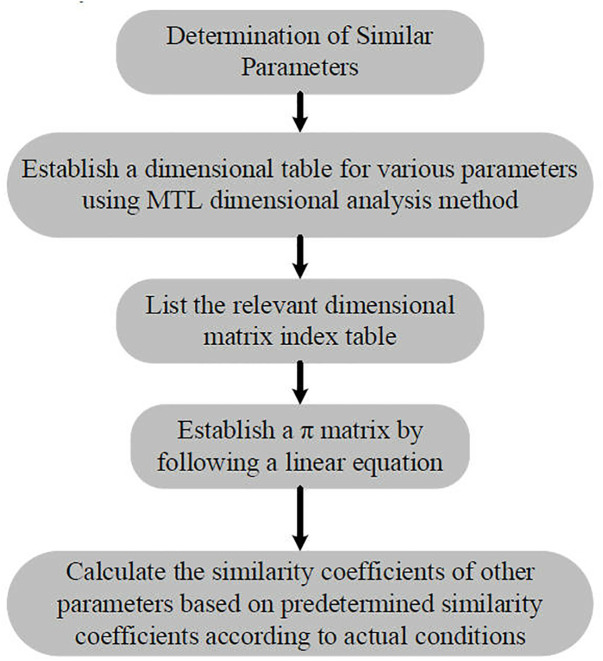
Technical route as determined by similar parameters.

**Fig 33:**
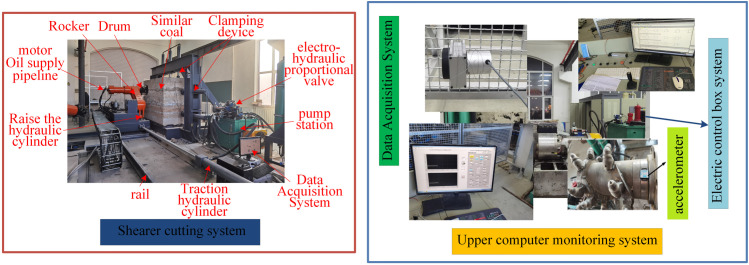
Self-adaptive height adjustment test system platform.

To accurately simulate the actual cutting process of the shearer prototype, the test model and coal wall construction must adhere to similarity criteria. In the derivation of these criteria, both the structural and kinematic parameters of the shearer, as well as the physical and mechanical properties of the coal wall, must be similar. Based on the technical route outlined in Fig 32, Using the MG2 × 55/250-BWD thin-seam shearer as the prototype, and guided by the principles of similarity theory, parameters such as drum diameter, drum speed, traction speed, force, torque, cutting power, vibration acceleration, density, and strength are selected as similarity parameters. Using the Mass Length Time(MLT) dimensional analysis method and the second similarity theorem, the similarity coefficients for the test rig and coal wall are determined, as shown in Table 12. Based on this, a comprehensive test rig for adaptive cutting control with a geometric similarity ratio of 1:2 is constructed, as shown in Fig 33.

A measurement and control system for the upper computer is established based on LabVIEW. By utilizing hybrid programming between LabVIEW and Matlab, the former calls the Simulink dynamic link library files. The PLC control system is built using OPC technology, ensuring communication between the PLC control system and the LabVIEW measurement and control system. The complete measurement and control system is then assembled. Using this experimental setup, a comparison is made to evaluate the superiority of five control strategies-fuzzy PID, DDPG, SAC, DQN, and TD3-in implementing hydraulic height adjustment for the shearer.

**Fig 34:**
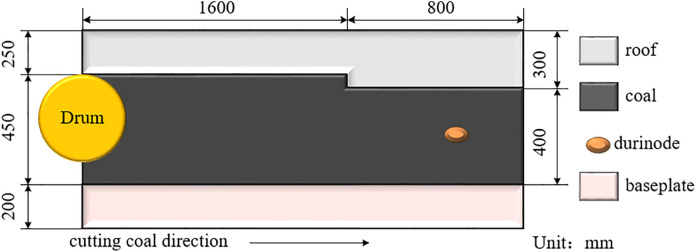
Simulated coal wall model.

The physical experiment is conducted under typical operating condition 1, where durinode are introduced. A coal wall model is constructed to match the mechanical properties of the 4602 working face at Yangcun Coal Mine, Yanzhou Coal Mining Group, as shown in Fig 34. The consistency of the mechanical properties of the coal wall model is validated using uniaxial compression tests.

**Fig 35:**
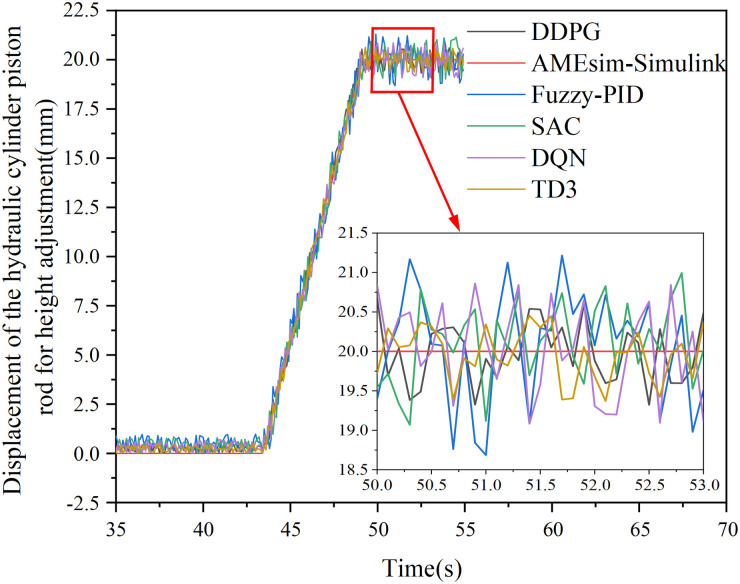
Hydraulic cylinder piston displacement test results and back deduction results data.

Based on the optimal drum speed (90 r/min) and traction speed (4.5 m/min) of the MG2 × 55/250-BWD thin -seam shearer, and using the similarity coefficients in Table 17, the drum speed was set to 108 r/min and the traction speed to 2.7 m/min for the simulated cutting test. The displacement data of the hydraulic cylinder piston rod was then extracted, and the experimental results were back-calculated using similarity principles. These results, shown in Fig 35, were compared with the results from the AMEsim-Simulink coupled simulation, yielding the maximum error, as presented in Table 18.

**Table 17:** Similarity coefficient of test bench and coal wall.

Parameter	Unit	Prototype	Similar model	Parameter	Unit	Prototype	Similar model
Drum diameter	mm	*D*	*D/2*	Force	N	*F*	0.09*F*
Drum speed	r/min	*n*	1.2*n*	Torque	N/m	T	0.045*T*
Traction speed	m/min	$v_{q}$	0.6*v*_*q*_	Cutting power	Kw	*P*	0.054 *P*
Density	$kg/m^{\text{3}}$	ρ	ρ	Vibration acceleration	$m/s^{\text{2}}$	*a*	0.72 *a*
Strength	MPa	σ	0.36*σ*	Time	s	t	0.83t

**Table 18:** Simulink mean error of model simulation and test platform test results.

Comparison and Analysis of Experimental Results	DDPG	Fuzzy PID	SAC	DQN	TD3
The displacement limit of the hydraulic cylinder piston rod during the simulation(mm)	20.0001	20.0005	20.0005	20.0002	20.0005
The displacement limit of the hydraulic cylinder piston rod during the experimental back-calculation(mm)	19.3325	18.6853	21.1504	19.0834	19.3586
The maximum relative error between the simulation and experimental back-calculation results(%)	3.33	6.58	5.75	4.58	3.21

From the error results in Table 18, the maximum relative error between the AMEsim-Simulink simulation results and the experimental back-calculation is 6.58% when using the Fuzzy PID controller, while the minimum relative error is 3.21% with the TD3 controller and 3.33% with the DDPG controller. These findings indicate that the five adaptive height control strategies for the shearer can accurately control the physical prototype. Moreover, they further validate the superiority of the DDPG control strategy over classical control algorithms and other typical deep reinforcement learning algorithms.

### 6.2 Analysis of error sources and uncertainty quantification

To further verify the stability of the AlexNet transfer learning model and the reliability of the experimental results, five repeated tests were conducted on the hoisting system controlled by the AlexNet transfer learning model (based on Typical Condition 1). The simulation and experimentally inferred errors of the hydraulic cylinder piston rod displacement were recorded, and the results are presented in Table 19.

**Table 19:** Verification of the stability and reliability of experimental results for the AlexNet transfer learning model.

Number of trials	Simulated value (mm)	Experimentally inferred value (mm)	Absolute error (mm)	Relative error (%)
1	20.0001	19.3325	0.6676	3.33
2	20.0001	19.4102	0.5899	2.95
3	20.0001	19.2876	0.7125	3.56
4	20.0001	19.3751	0.6250	3.12
5	20.0001	19.4528	0.5473	2.74
Statistical value			Mean = 0.6285	Mean = 3.14
			Standard deviation = 0.0621	Standard deviation = 0.29

Based on the results of five repeated tests in Table 19, the mean relative error between the simulation and experimentally inferred results is 3.14%, with a standard deviation of 0.29% and a coefficient of variation of 9.2%. These values indicate a concentrated error distribution and demonstrate the good stability of the experimental results. Considering the characteristics of the experimental system, the main sources of error are as follows: (1) measurement errors from displacement sensors, data acquisition cards, and other measurement devices; (2) discrepancies between the simulated coal wall and the actual physical and mechanical properties of the coal seam; and (3) scale effect errors arising from the similitude physical test bench.

## 7 Conclusion

In response to the challenges faced by classical optimization control algorithms in achieving self-adaptive control for the hydraulic height adjustment of shearer drums, as well as the slow response and poor tracking performance of certain typical deep reinforcement learning algorithms, this study focuses on the MG2 × 55/250-BWD shearer and the coal seams of the 4602 working face at Yangcun Coal Mine, Yanzhou Coal Mining Group. The study introduces a method for distinguishing coal-rock cutting states based on a DDPG algorithm, using the SVD-CWT technique to denoise the drum vibration acceleration signals, which are then transformed into time-frequency spectrograms and input into the AlexNet transfer learning model. The following conclusions can be drawn from the simulations and physical experiments comparing the proposed DDPG-based control strategy with classical control algorithms and other typical DRL algorithms:

(1) The drum vibration acceleration signals were denoised using SVD-CWT, and then converted into time-frequency spectrograms. The significant differences in energy distribution and intensity allowed for effective differentiation of coal-rock cutting states. These spectrograms were used as input to a trained AlexNet transfer learning model, achieving a recognition accuracy of 95.06%.
(2) By simulating the continuous and sudden displacement changes of the hydraulic cylinder’s piston rod with sine and square wave signals, the system exhibits tracking lag times of 0.08s and 0.158s, respectively, demonstrating good tracking performance. After being subjected to disturbance, the system returns to a stable state in just 0.13s, indicating strong robustness against external disturbance signals. The system’s environmental self-adaptability is further analyzed by simulating different working conditions with step signals of varying amplitudes. The maximum difference in the time required to reach steady state is only 0.007s, and the maximum steady-state error is just$9\times 10^{-4}mm$, confirming the system’s strong environmental self-adaptability.
(3) The self-adaptive height control system based on the DDPG algorithm outperformed the classical control systems in several aspects. The response time was reduced by up to 0.163s, the steady-state error decreased by 0.47948 mm, and the time required to return to steady state after external disturbances was shortened by up to 0.75s. Compared to the TD3 algorithm, the DDPG-based system showed reductions in rise time and adjustment time by 121.95% and 62.35%, respectively. When compared to the SAC algorithm, the DDPG-based system reduced the steady-state error from 0.0824 mm to 0.0005 mm. These results indicate that the DDPG algorithm better meets the requirements for environmental self-adaptability, fast system response, stability, and accuracy, with superior disturbance rejection capability.
(4) The feasibility of the proposed control strategy was also validated through AMEsim-Simulink co-simulations. Compared to the TD3 algorithm, the DDPG algorithm resulted in an 84.95% reduction in adjustment time, and when compared to the SAC algorithm, the steady-state error was reduced from 0.1027 mm to 0.0021 mm. This highlights the DDPG controller’s ability to adapt effectively to the uncertainties associated with complex coal seam conditions. Physical experiments confirmed that the proposed self-adaptive control strategy could accurately control the physical prototype, with the mean of maximum error between the simulation and experimental results being only 3.14%. Simultaneously, a quantitative analysis of the uncertainty was performed. The results indicate that the standard deviation between the simulation and experimentally inferred results is 0.29%, with a coefficient of variation of 9.2%. These values demonstrate a concentrated error distribution and confirm the good stability of the experimental results. These findings further validate the superiority of the DDPG control strategy, offering a novel approach for achieving precise adaptive height adjustment in shearers.

## Supporting information

S1 Data Basic data. (DOCX)

S2 Data Experimental result data. (XLS)
