# Supplementary material for: Research on self-adaptive height adjustment control of shearer based on deep deterministic policy gradient
Source: PLoS One. 2026 Jan 22;21(1):e0329347. doi: 10.1371/journal.pone.0329347 (PMC12826524; doi:10.1371/journal.pone.0329347)
Supplement: S1 Data — (DOCX) [file pone.0329347.s001.docx]

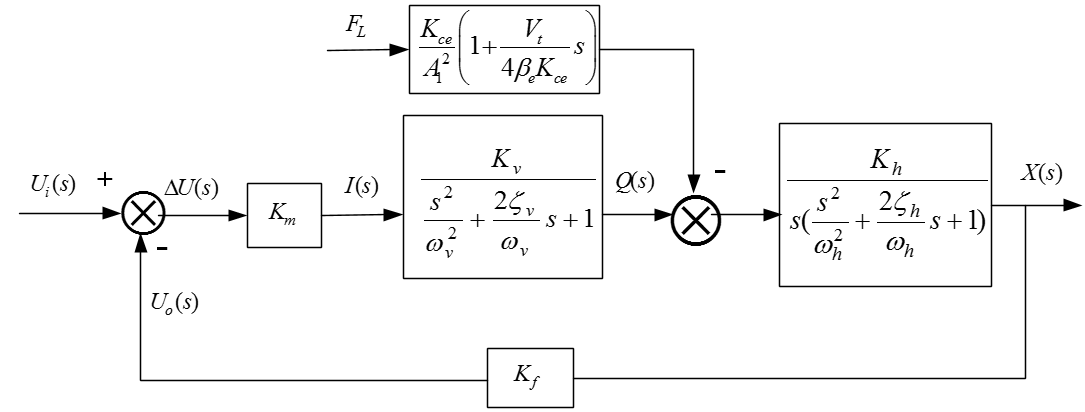


Fig1 Transfer function block diagram of the self-adaptive height adjustment control system

Table 1 Simulation parameters

| Parameter | Value |
| --- | --- |
| $A_{1}$ | $2.0106\times10^{-2}m^{2}$ |
| $A_{2}$ | $1.2252\times10^{-2}m^{2}$ |
| $\zeta_{h}$ | 0.7 |
| $K_{m}$ | 2.25AV^-1^ |
| $K_{f}$ | 6.56Vm^-1^ |
| $K_{v}$ | $4.8\times10^{-4}m^{3}s^{-1}A^{-1}$ |
| $\omega_{v}$ | 157rads^-1^ |
| $\zeta_{v}$ | 0.2 |
| $K_{ce}$ |  |
| $\beta_{e}$ | $0.9\times10^{9}Pa^{2}$ |
| $F_{L}$ | 5 kN |
| $\omega_{h}$ | 127.2rads^-1^ |
| $\zeta_{h}$ | 0.1 |
| $V_{t}$ | $0.1803\times10^{-2}m^{3}$ |


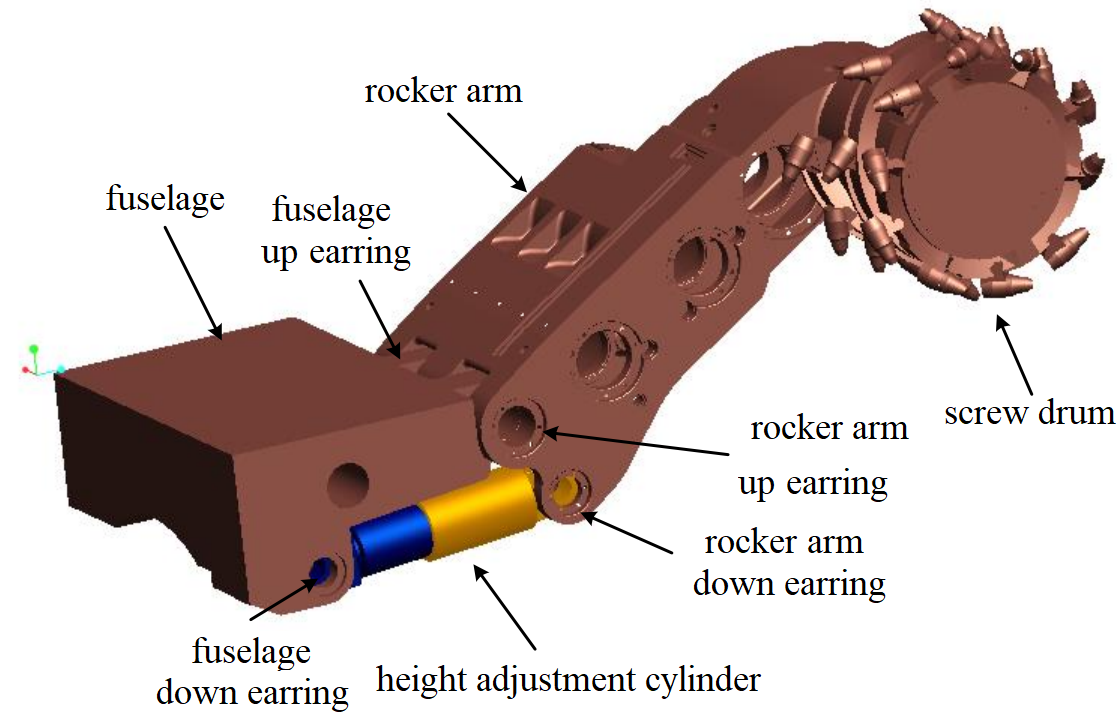


Fig 2 3D solid model of shearer height adjustment mechanism

Table 2 The main structural parameter values of the shearer and drum

| Drum Parameter | Value | Unit | Drum Parameter | Value | Unit |
| --- | --- | --- | --- | --- | --- |
| Drum Diameter | 800 | mm | Drum Hub Outer Diameter | 465 | mm |
| Spiral Blade Height | 68 | mm | Drum Hub Inner Diameter | 425 | mm |
| Spiral Blade Thickness | 90 | mm | Tooth Arrangement Type | Sequential |  |
| Spiral Blade Pitch Angle | 14 | ° | Drum Cutting Depth | 630 | mm |
| Number of Spiral Blades | 2 |  | Number of Teeth per Blade Line | 2 |  |
| Length of Small Rock Arm | | | | 260 | mm |
| Length of Drum Rock Arm | | | | 1400 | mm |
| Distance from Lower Pivot of the Body to Upper Pivot of Rock Arm | | | | 712 | mm |
| Distance from Drum's Lowest Point to Hydraulic Cylinder Pivot | | | | 635 | mm |

Table 3 Typical working conditions

| Condition Number | Coal Wall Type | Hydraulic Cylinder Piston Retraction Distance (mm) | Spiral Drum Corresponding Downward Adjustment Height (mm) |
| --- | --- | --- | --- |
| 1 | Coal→Roof + Coal | 20 | 100 |
| 2 | Coal→Roof + Coal | 40 | 214 |
| 3 | Coal→Roof + Coal | 60 | 325 |
| 4 | Coal→Roof + Coal + Durinode | 50 | 270 |


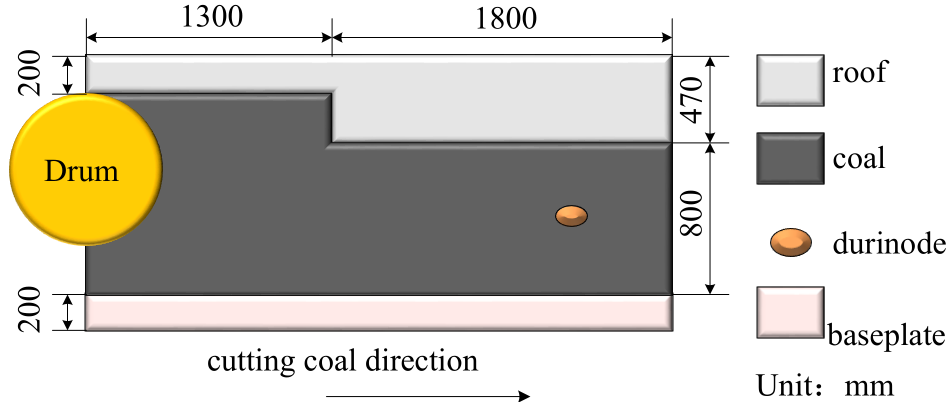


Fig 3 Coal wall model of working condition 4

Table 4 Contact parameters between particles

| mutually contacting particle type | Coefficient of Restitution | Coefficient of Static Friction | Coefficient of Kinetic Friction |
| --- | --- | --- | --- |
| Coal & Coal | 0.58 | 0.62 | 0.082 |
| Coal & Gangue | 0.41 | 0.46 | 0.128 |
| Coal & Roof | 0.42 | 0.44 | 0.134 |
| Gangue & Gangue | 0.47 | 0.50 | 0.113 |
| Roof & Roof | 0.45 | 0.48 | 0.109 |

Table 5 Mechanical parameters of bonding between particles

| Mutually Bonded Particle Type | Normal Stiffness(N/m^3^) | Tangential Stiffness(N/m^3^) | Maximum Normal Stress(Pa) | Maximum Tangential Stress(Pa) |
| --- | --- | --- | --- | --- |
| Coal & Coal | 1.3008×10^8^ | 1.0104×10^8^ | 7.7841×10^6^ | 2.0147×10^6^ |
| Coal & Gangue | 1.9537×10^8^ | 1.5629×10^8^ | 1.7615×10^7^ | 7.4213×10^6^ |
| Coal & Roof | 2.3017×10^8^ | 1.8415×10^8^ | 1.8543×10^7^ | 7.3865×10^6^ |
| Gangue & Gangue | 7.4136×10^8^ | 5.9309×10^8^ | 2.7563×10^7^ | 1.2793×10^7^ |
| Roof & Roof | 2.2017×10^9^ | 1.8775×10^9^ | 2.8936×10^7^ | 1.2174×10^7^ |

Fig 4 Vibration acceleration curves of the spiral drum under cutting conditions of *f*=3.5 coal and *f*=3.5 Roof +Coal

Table 6 The signal characteristic value of vibration of drum X, Y and Z

| Coal-Rock Type | Direction | Vibration Acceleration Time-Domain Signal Features | | | |
| --- | --- | --- | --- | --- | --- |
|  |  | Maximum Value (mm/s²) | Minimum Value (mm/s²) | Peak Value (mm/s²) | Root Mean Square (RMS) Value (mm/s²) |
| Coal | X | 30833.40 | -27985.46 | 6093.81 | 7839.54 |
|  | Y | 15413.99 | -16024.58 | 4095.37 | 1687.04 |
|  | Z | 39874.25 | -38761.63 | 9764.92 | 3782.99 |
| Roof+Coal | X | 31587.43 | -28739.32 | 5883.71 | 7562.82 |
|  | Y | 15921.43 | -16239.43 | 3902.43 | 1617.32 |
|  | Z | 40195.22 | -39758.31 | 9597.41 | 3654.28 |

Table 7 Parameter setting of CWT

| Parameter | Parameter selection |
| --- | --- |
| Wavelet Basis | cmor |
| Bandwidth | 1 |
| Center frequency | 100 |
| Scale | 300 |
| Pixel | 227*227 |
| Boundary treatment | symmetric extension |


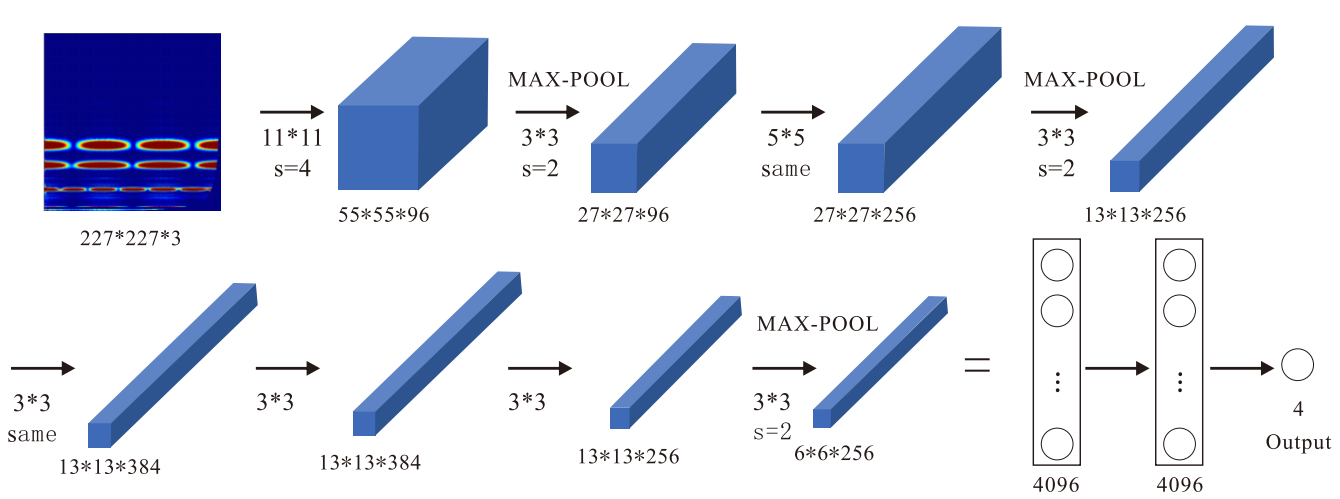


Fig 5 AlexNet transfer learning model

Table 8 Main parameter assignment

| Layer Structure | Input Channels | Output Channels | Input Feature  Map Size | Output Feature Map Size | Kernel/Pool Size | Stride | Padding Value |
| --- | --- | --- | --- | --- | --- | --- | --- |
| Conv1 | 3 | 96 | 227×227 | 55×55 | 11×11 | 4 | 0 |
| Max Pooling1 | 96 | 96 | 55×55 | 27×27 | 3×3 | 2 | 0 |
| Conv2 | 96 | 256 | 27×27 | 27×27 | 5×5 | 1 | 2 |
| Max Pooling2 | 256 | 256 | 27×27 | 13×13 | 3×3 | 2 | 0 |
| Conv3 | 256 | 384 | 13×13 | 13×13 | 3×3 | 1 | 1 |
| Conv4 | 384 | 384 | 13×13 | 13×13 | 3×3 | 1 | 1 |
| Conv5 | 384 | 256 | 13×13 | 13×13 | 3×3 | 1 | 1 |
| Max Pooling3 | 256 | 256 | 13×13 | 6×6 | 3×3 | 2 | 0 |

Table.9 Parameter setting of deep neural network

| Network Parameters  Critic/Actor | Learning Rate | Gradient Threshold Method | Gradient Threshold |
| --- | --- | --- | --- |
|  | $1\times10^{-4}$ | l2norm | 1 |
|  | Optimizer | L2 Regularization Facto | Use Device |
|  | Adam | $1\times10^{-4}$ | CPU |

Table.10 Agent parameter settings

| Agent  Parameters | Target Network  Update Method | Target Network Update Delay Factor | Target Network Update Frequency | Noise Mechanism | |
| --- | --- | --- | --- | --- | --- |
|  |  |  |  | Variance | Variance Decay Rate |
|  | Smoothing | $1\times10^{-3}$ | 1 | 0.5 | $1\times10^{-5}$ |
|  | Sampling Time | Reward Discount Factor | Batch Size | Experience Replay  Pool Size | |
|  | 0.05s | 0.9 | 64 | $1\times10^{5}$ | |
